# Supplementary material for: Immune stress suppresses innate immune signaling in preleukemic precursor B-cells to provoke leukemia in predisposed mice
Source: Nat Commun. 2023 Aug 24;14:5159. doi: 10.1038/s41467-023-40961-z (PMC10449887; doi:10.1038/s41467-023-40961-z)
Supplement: Supplementary file 1 — Supplementary Information [file 41467_2023_40961_MOESM1_ESM.pdf]

SUPPLEMENTARY INFORMATION for

Immune stress suppresses innate immune signaling in preleukemic precursor B cells to provoke leukemia in predisposed mice

Marta Isidro-Hernández M, et al.

SUPPLEMENTARY FIGURES:

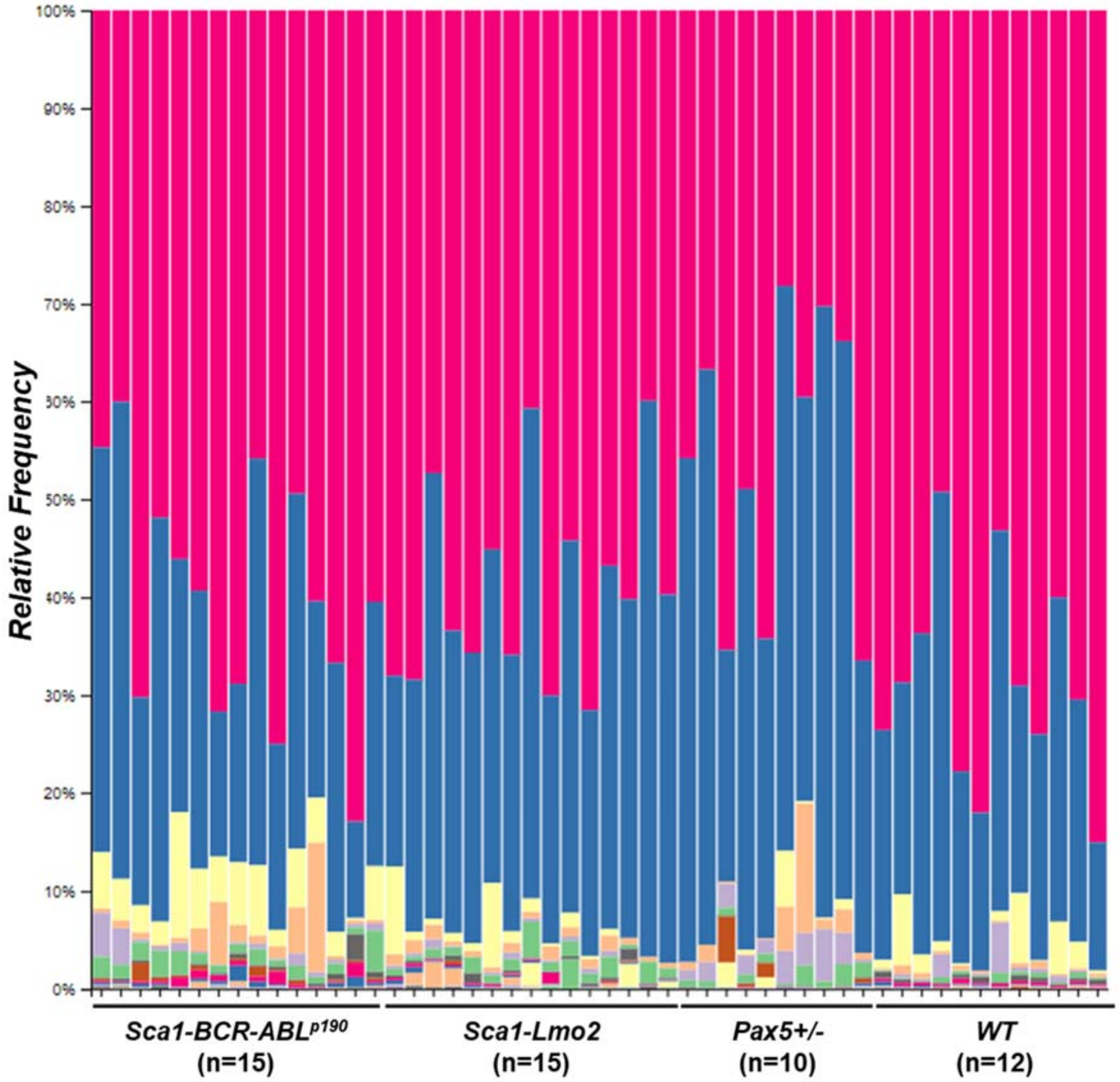

Legend:

D_0__Bacteria;D_1__Bacteroidetes;D_2__Bacteroidia;D_3__Bacteroidales
D_0__Bacteria;D_1__Firmicutes;D_2__Clostridia;D_3__Clostridiales
D_0__Bacteria;D_1__Tenericutes;D_2__Mollicutes;D_3__Anaeroplasmatales
D_0__Bacteria;D_1__Firmicutes;D_2__Bacilli;D_3__Lactobacillales
D_0__Bacteria;D_1__Deferribacteres;D_2__Deferribacteres;D_3__Deferribacterales
D_0__Bacteria;D_1__Proteobacteria;D_2__Deltaproteobacteria;D_3__Desulfovibrionales
D_0__Bacteria;D_1__Proteobacteria;D_2__Gammaproteobacteria;D_3__Betaproteobacteriales
D_0__Bacteria;D_1__Cyanobacteria;D_2__Melainabacteria;D_3__Gastranaerophilales
D_0__Bacteria;D_1__Patescibacteria;D_2__Saccharimonadia;D_3__Saccharimonadales
D_0__Bacteria;D_1__Firmicutes;D_2__Erysipelotrichia;D_3__Erysipelotrichales
D_0__Bacteria;D_1__Verrucomicrobia;D_2__Verrucomicrobiae;D_3__Verrucomicrobiales
D_0__Bacteria;D_1__Actinobacteria;D_2__Actinobacteria;D_3__Bifidobacteriales
D_0__Bacteria;D_1__Proteobacteria;D_2__Alphaproteobacteria;D_3__Rhodospirillales
D_0__Bacteria;D_1__Proteobacteria;D_2__Gammaproteobacteria;D_3__Enterobacteriales
D_0__Bacteria;D_1__Actinobacteria;D_2__Coriobacteriia;D_3__Coriobacteriales
D_0__Bacteria;D_1__Proteobacteria;D_2__Gammaproteobacteria;D_3__Xanthomonadales
D_0__Bacteria;__;__;__
D_0__Bacteria;D_1__Proteobacteria;D_2__Alphaproteobacteria;D_3__Rickettsiales
D_0__Bacteria;D_1__Tenericutes;D_2__Mollicutes;D_3__Mollicutes RF39
D_0__Bacteria;D_1__Cyanobacteria;D_2__Oxyphotobacteria;D_3__Chloroplast
Unassigned;__;__;__
D_0__Bacteria;D_1__Firmicutes;D_2__Bacilli;D_3__Bacillales
D_0__Bacteria;D_1__Proteobacteria;D_2__Gammaproteobacteria;D_3__Pseudomonadales
D_0__Bacteria;D_1__Firmicutes;D_2__Negativicutes;D_3__Selenomonadales
D_0__Bacteria;D_1__Actinobacteria;D_2__Actinobacteria;D_3__Micrococcales
D_0__Bacteria;D_1__Proteobacteria;D_2__Alphaproteobacteria;D_3__Rhizobiales
D_0__Bacteria;D_1__Actinobacteria;D_2__Actinobacteria;D_3__Corynebacteriales
D_0__Bacteria;D_1__Actinobacteria;D_2__Actinobacteria;D_3__Propionibacteriales
D_0__Bacteria;D_1__Fusobacteria;D_2__Fusobacteriia;D_3__Fusobacteriales
D_0__Bacteria;D_1__Bacteroidetes;D_2__Bacteroidia;D_3__Chitinophagales
D_0__Bacteria;D_1__Acidobacteria;D_2__Acidobacteriia;D_3__Acidobacteriales
D_0__Bacteria;D_1__Verrucomicrobia;D_2__Verrucomicrobiae;D_3__Chthoniobacterales
D_0__Bacteria;D_1__Deinococcus-Thermus;D_2__Deinococci;D_3__Deinococcales
D_0__Bacteria;D_1__Acidobacteria;D_2__Acidobacteriia;D_3__Subgroup 2
D_0__Bacteria;D_1__Planctomycetes;D_2__Phycisphaerae;D_3__Tepidisphaerales

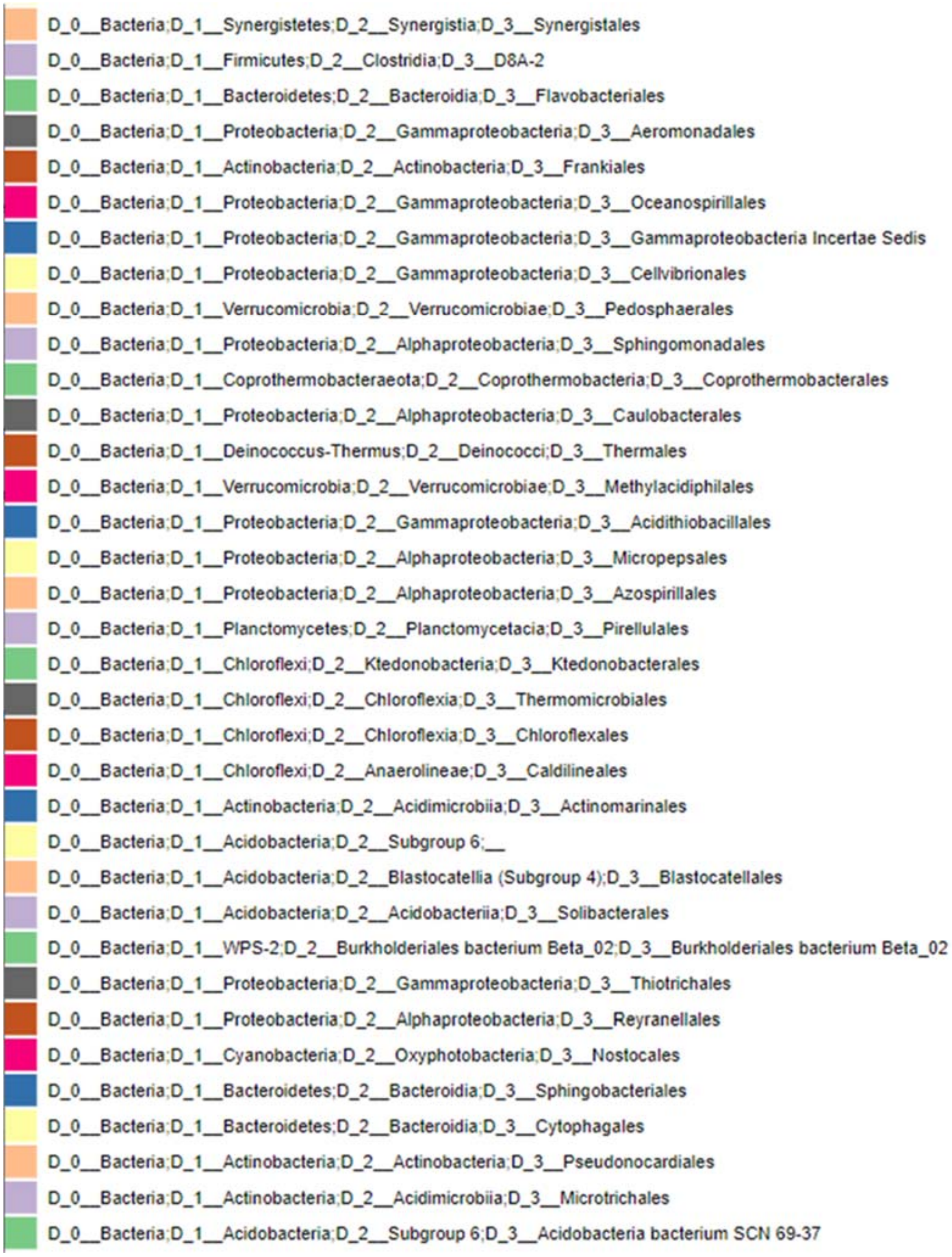

Supplementary Fig. S1: Gut microbiome analysis of *Sca1-BCR-ABLp190*, *Sca1-Lmo2*, *Pax5*$^{+/-}$ and WT mice housed in a pathogen-free facility. Taxa Bar plot of the Microbial V4-16S signatures (V4-ASVs) grouped by mouse genotype (*Sca1-BCR-ABLp190* n=15, *Sca1-Lmo2* n=15, *Pax5*$^{+/-}$ n=10 and WT n=12) using 4 taxonomic levels. The legend lists the categorical Taxon orderer from more (top) to less (bottom) frequency.

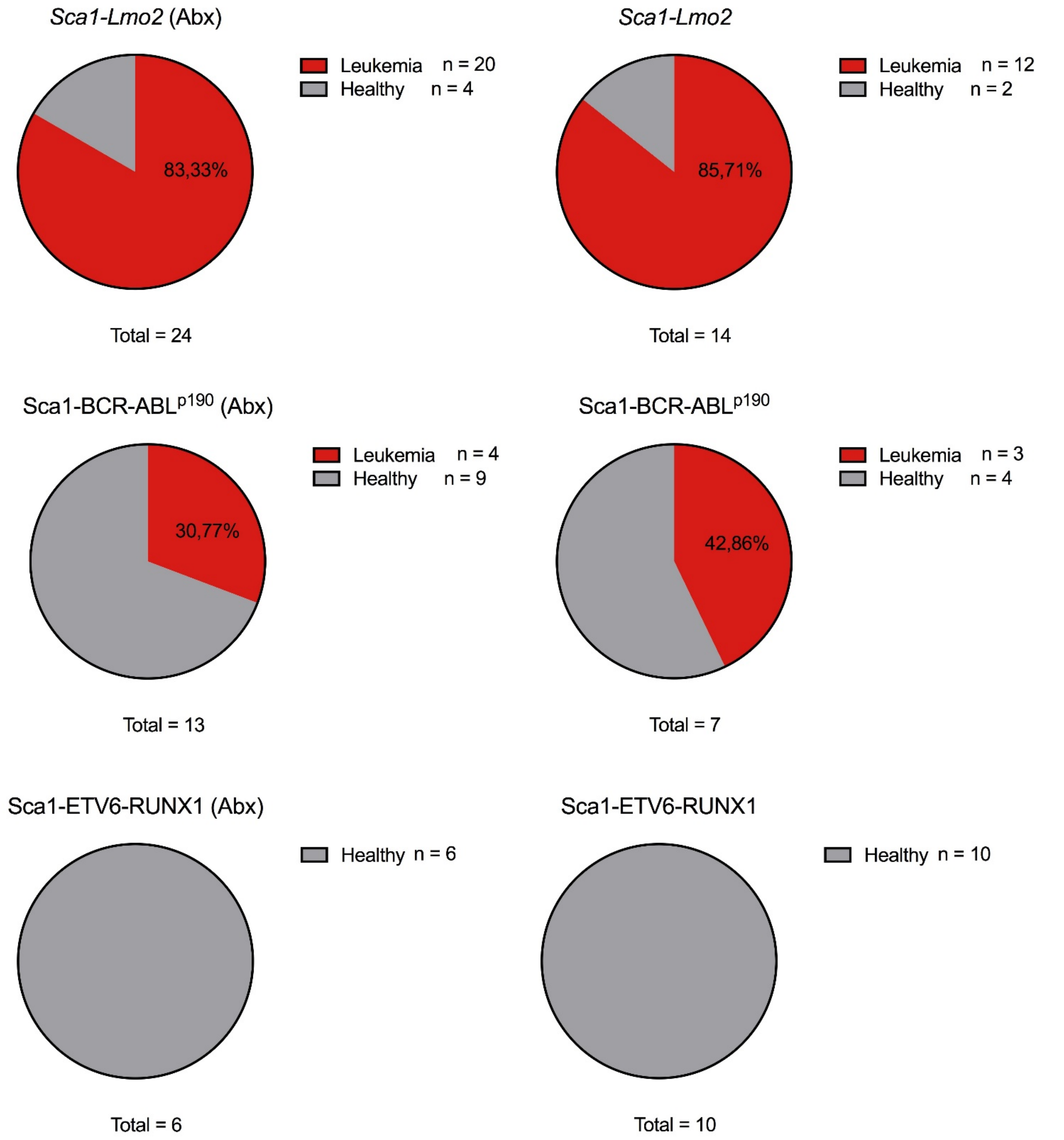

Supplementary Fig. S2: Leukemia incidence of *Sca1-BCR-ABLp190*, *Sca1-Lmo2*, and *Sca1-ETV6-RUNX1* mice with and without gut microbiota deprivation. All the mice were housed in a pathogen-free facility and some of the *Sca1-BCR-ABLp190, Sca1-Lmo2*, and *Sca1-ETV6-RUNX1* mice were treated with a cocktail of antibiotics (Abx) for 8 weeks to induce the alteration of the gut microbiome. No significant differences were found using Fisher's exact test. In all, none of the microbiome-deprived *Sca1-ETV6-RUNX1* mice (n=6) developed B-ALL; 30.77% (4/13) of *Sca1-BCR-ABLp190* treated with an antibiotic developed B-ALL and 83.33% (16/24) of the microbiome-deprived *Sca1-Lmo2* developed T-ALL. *Sca1-ETV6-RUNX1* mice need exposure to infection to develop B-ALL, for this reason, none of the mice developed B-ALL under these housing conditions. Source data are provided as a Source Data file.

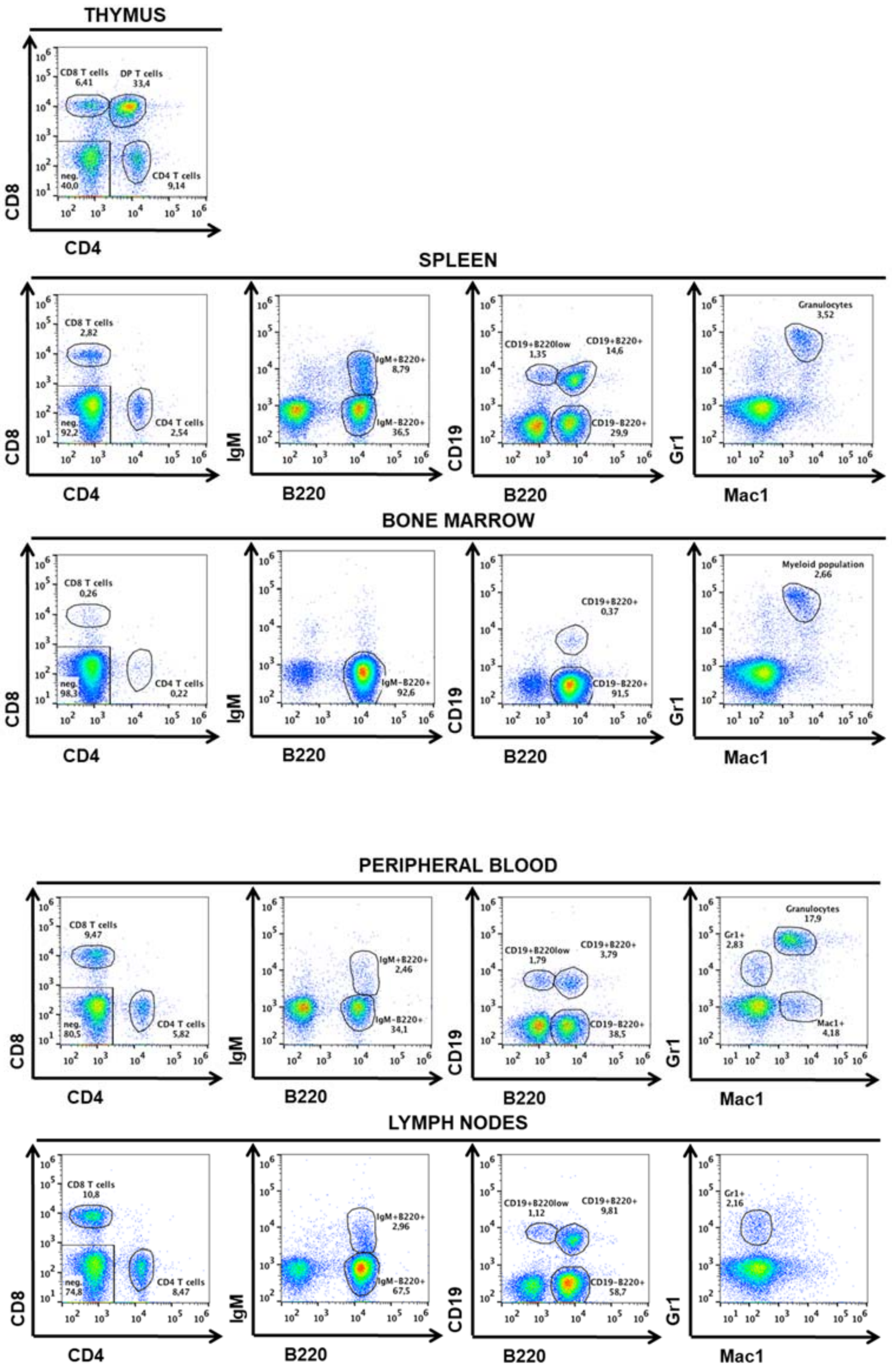

Supplementary Fig. S3: Flow cytometric analysis of hematopoietic subsets in diseased *Pax5*$^{+/-}$ early-exposure mice. Representative plots of cell subsets from the thymus, spleen, bone marrow (BM), peripheral blood (PB), and lymph nodes (LN) are shown from a diseased *Pax5*$^{+/-}$ early-exposure mouse. A total of 7 diseased mice were analyzed by flow cytometry (age: 9-17 months). FACS analysis revealed a cell surface phenotype CD19$^{+/-}$B220^{+}IgM^{-} for tumor cells that extended through BM, PB, spleen, and LN.

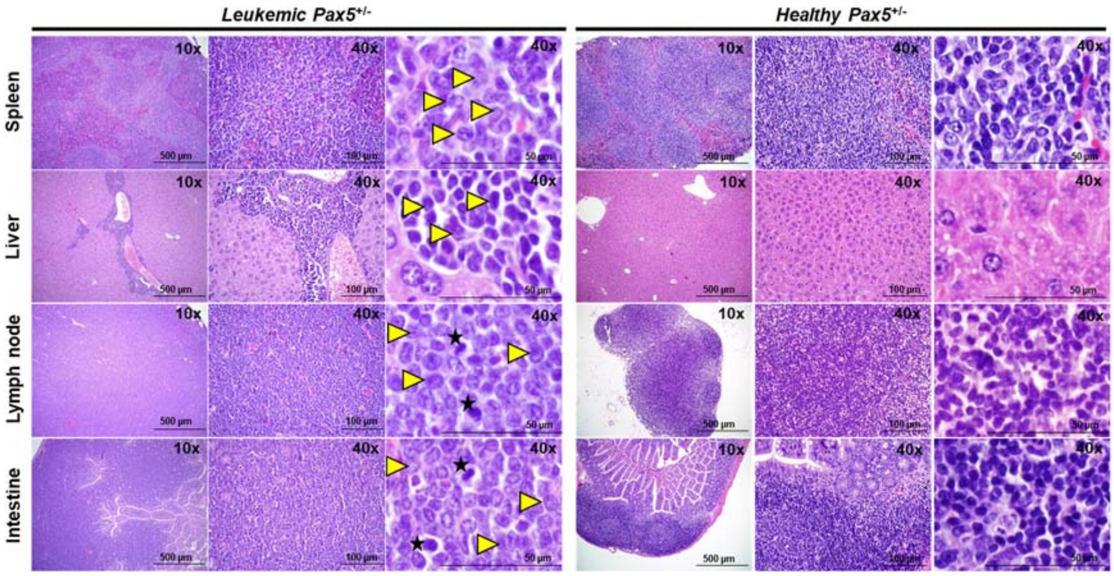

Supplementary Fig. S4: B-ALL in *Pax5*$^{+/-}$ mice early exposed to common infections. Haematoxylin and eosin staining of a *Pax5*$^{+/-}$ (X005) mouse housed in SPF conditions and a tumour-bearing *Pax5*$^{+/-}$ early-exposure mouse (R324) showing infiltrating blast cells in spleen, liver, lymph node, and intestine. Loss of normal architecture resulting with cells morphologically resembling lymphoblasts can be shown (n=7). Tissues from a control littermate wild type mouse are shown for reference. Magnification and the corresponding scale bar are indicated in each case. Yellow arrows highlight some of the leukemic infiltrating cells within the tumor. Asterisks highlight leukemic cells in mitosis.

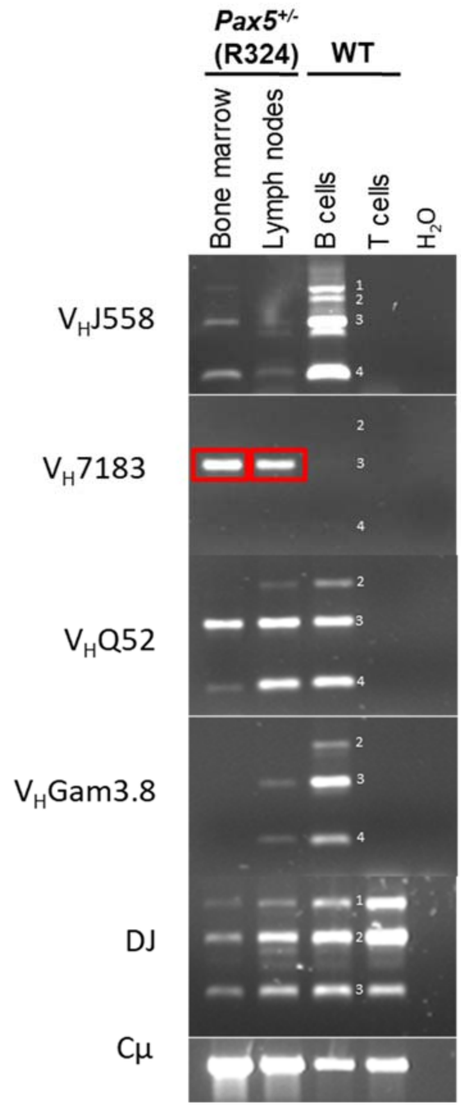

Supplementary Fig. S5: Analysis of BCR clonality of leukemia arising in a *Pax5*$^{+/-}$ mouse from the early-exposure group. PCR analysis of BCR gene rearrangements in the bone marrow and lymph nodes of a diseased mouse. Infiltrated tissues show increased clonality within their immunoglobulin repertoire (red square) (n=2). Sorted CD19^{+} splenic B cells (B cells) of healthy mice serve as a control for polyclonal BCR rearrangements (indicated by numbers, 1-4) and CD8^{+}CD4^{+} T cells from the thymus of healthy mice served as a negative control. Source data are provided as a Source Data file.

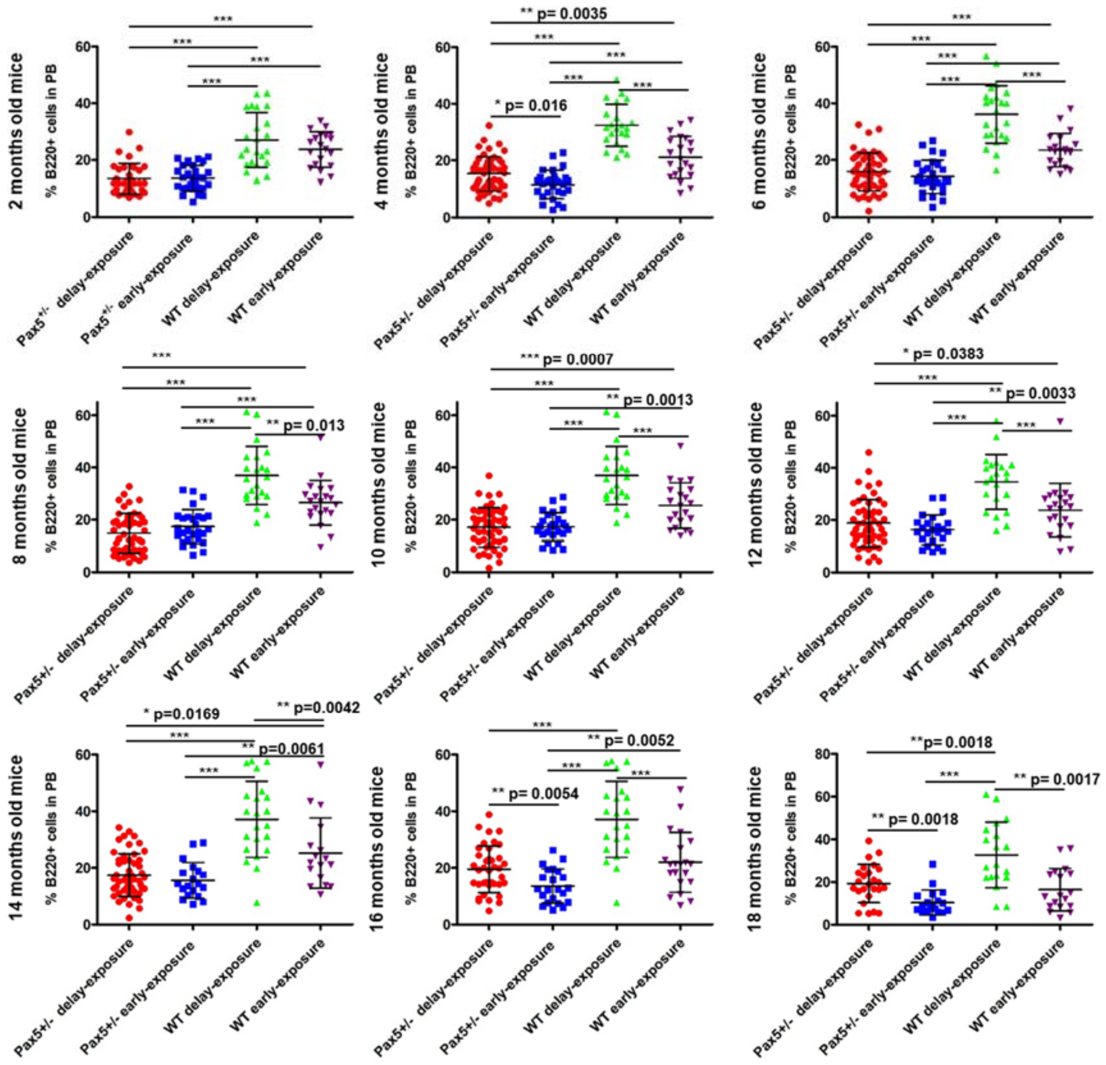

Supplementary Fig. S6: Peripheral B-cell decrease in *Pax5*$^{+/-}$ mice. Percentage of peripheral blood B-cells (B220^{+}IgM$^{+/-}$) at different time points in *Pax5*$^{+/-}$ early-exposure mice (n=19-28) compared to WT early-exposure mice (n=19-21), *Pax5*$^{+/-}$ delay-exposure mice (n=25-34) and WT delay-exposure mice (n=18-23) analyzed by flow cytometry. Error bars represent the standard deviation. Mann Whitney p-values are indicated in each case. (*** for p <0.0001). Source data are provided as a Source Data file.

	DNA sequencing project number	ID	mouse age at disease	CD19 phenotype	JAK3 Mutation in mouse	JAK3 Human homolog	Pax5 mutation of WT allele	Pax5 Human homolog	Other mutations
1	123BM	R324	10,4 months	CD19-	-	-	-	-	-
2	141BM	R330	12,03 months	CD19+	-	-	P80R	P80R	Kras Q61H
3	157BM	T830	9 months	CD19low	-	-	P80R	P80R	Jak1 F837V
4	158T	R017	17,23 months	CD19-	-	-	-	-	Nras G13D

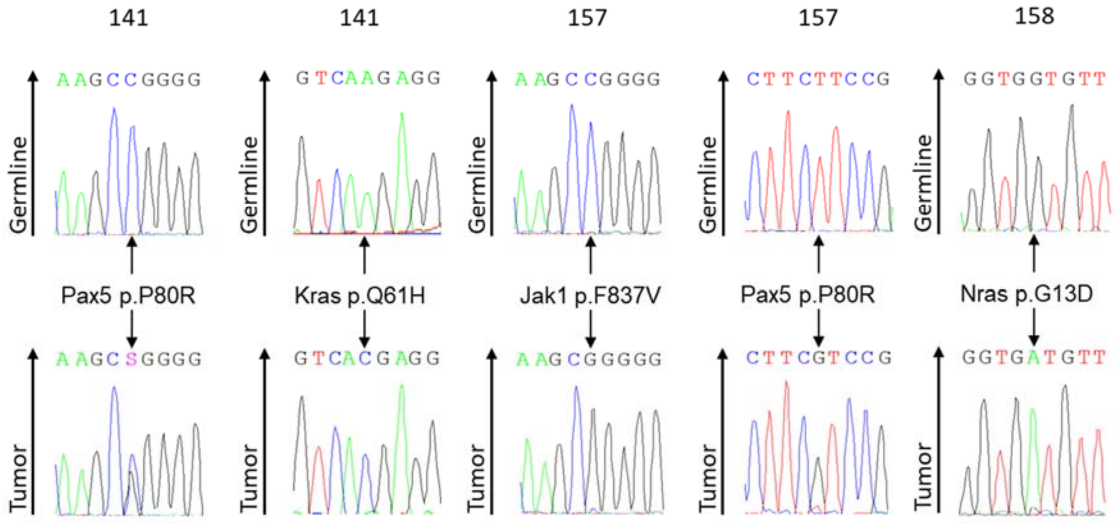

Supplementary Fig. S7: Secondary mutations arising in tumor-bearing *Pax5*$^{+/-}$ early-exposure mice. Whole exome sequencing (WES) of 4 *Pax5*$^{+/-}$ early-exposure tumors and corresponding germline was performed on a HiSeq 2500 (Illumina) platform. *Pax5*$^{+/-}$ early-exposure tumor DNA was derived from leukemic bone marrow, while tail DNA of the respective mouse was used as reference germline material. Likewise, *Pax5*$^{+/-}$ leukemias from the delayed group, *Pax5*$^{+/-}$ early-exposure tumors showed recurrent mutations affecting the remaining WT *Pax5* allele, with Pax5 p.P80R occurring in 2 out of 4 analyzed leukemias. All the variants found by WES were confirmed by Sanger sequencing.

a

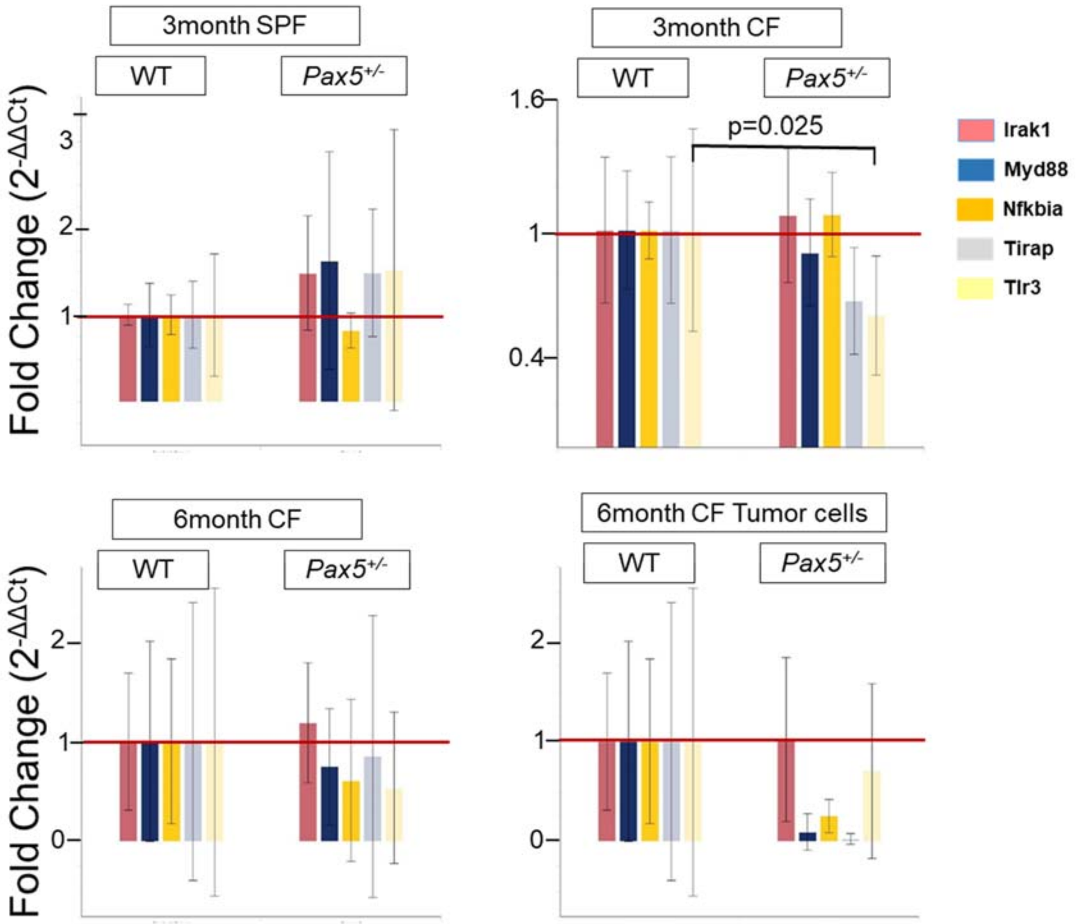
3month SPF
WT
Pax5+/-
Fold Change (2-ΔΔCt)
3
2
1
3month CF
WT
Pax5+/-
1.6
p=0.025
1
0.4
Irak1
Myd88
Nfkbia
Tirap
Tlr3
6month CF
WT
Pax5+/-
Fold Change (2-ΔΔCt)
2
1
0
6month CF Tumor cells
WT
Pax5+/-
2
1
0

b

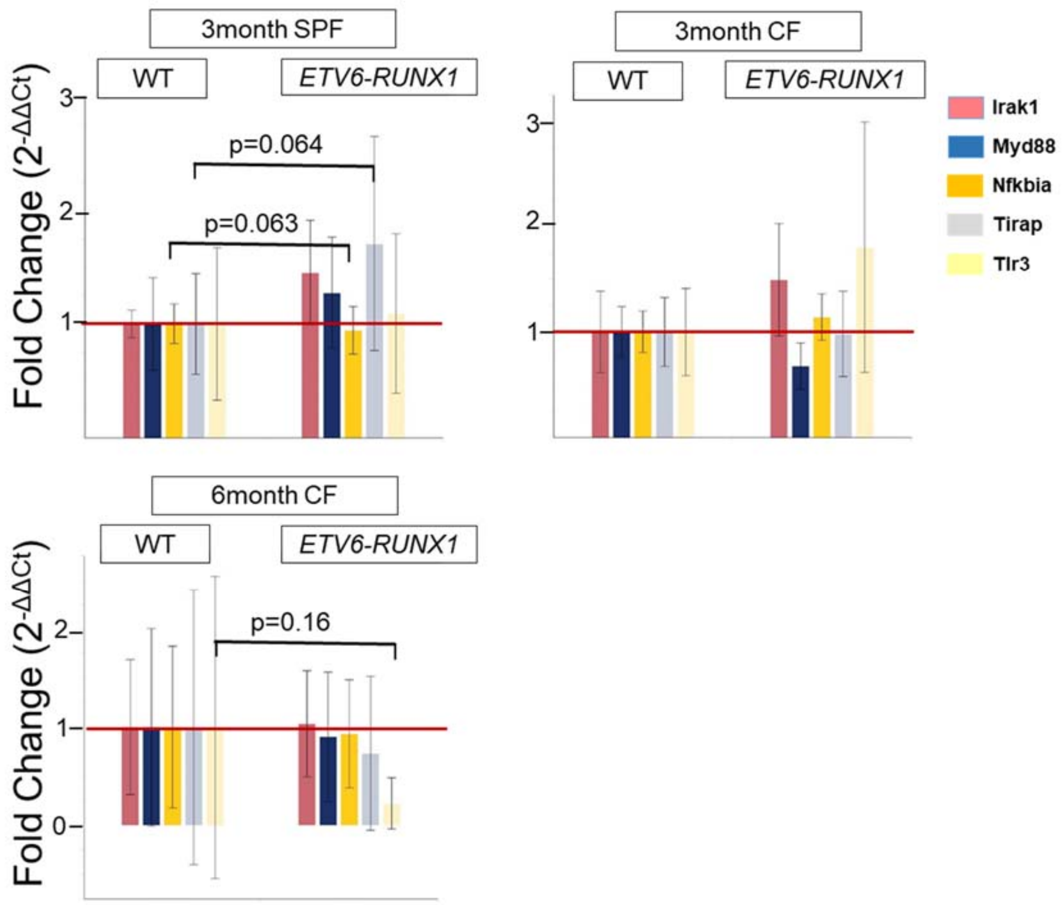

Supplementary Fig. S8: RT2 Profiler PCR Array Mouse Toll-Like Receptor Signaling Pathway. a) Sorted proB cells (B220^{low} IgM^{-}) obtained from WT (n= 4), healthy *Pax5*$^{+/-}$ (n=4), and leukemic *Pax5*$^{+/-}$ mice (n= 2) exposed to different environmental contexts (SPF, specific-pathogen facility and CF, conventional facility) were assessed by using Toll-like receptor signalling pathway PCR array. **b**) Sorted proB cells (B220^{low} IgM^{-}) obtained from WT (n= 4) and healthy *Sca1-ETV6-RUNX1* mice (n=4) exposed to different environmental contexts (SPF and CF facilities) were assessed by using the same Toll-like receptor signalling pathway PCR array. Data were represented as fold change (2-ΔΔCt) using the comparative CT Method. The p values are calculated based on a Student's t-test of the replicate 2^(- Delta CT) values for each gene. Source data are provided as a Source Data file.

a

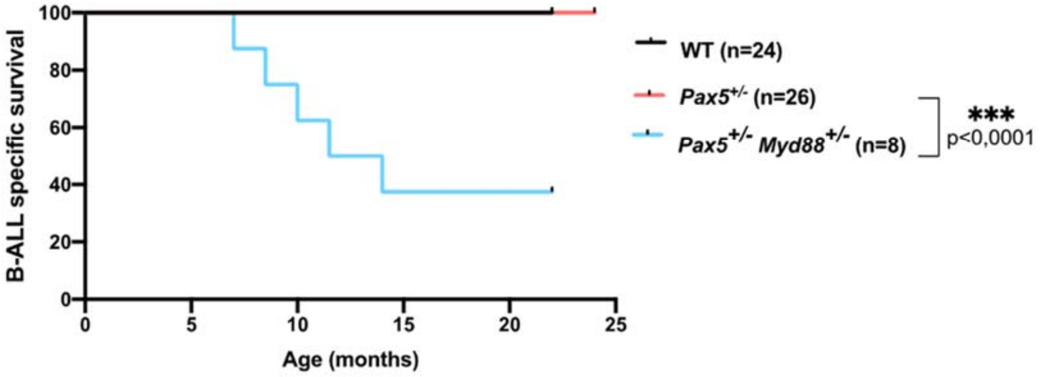

B-ALL specific survival
100
80
60
40
20
0
0
5
10
15
20
25
Age (months)
WT (n=24)
Pax5+/- (n=26)
Pax5+/- Myd88+/- (n=8)
***
p<0,0001

b

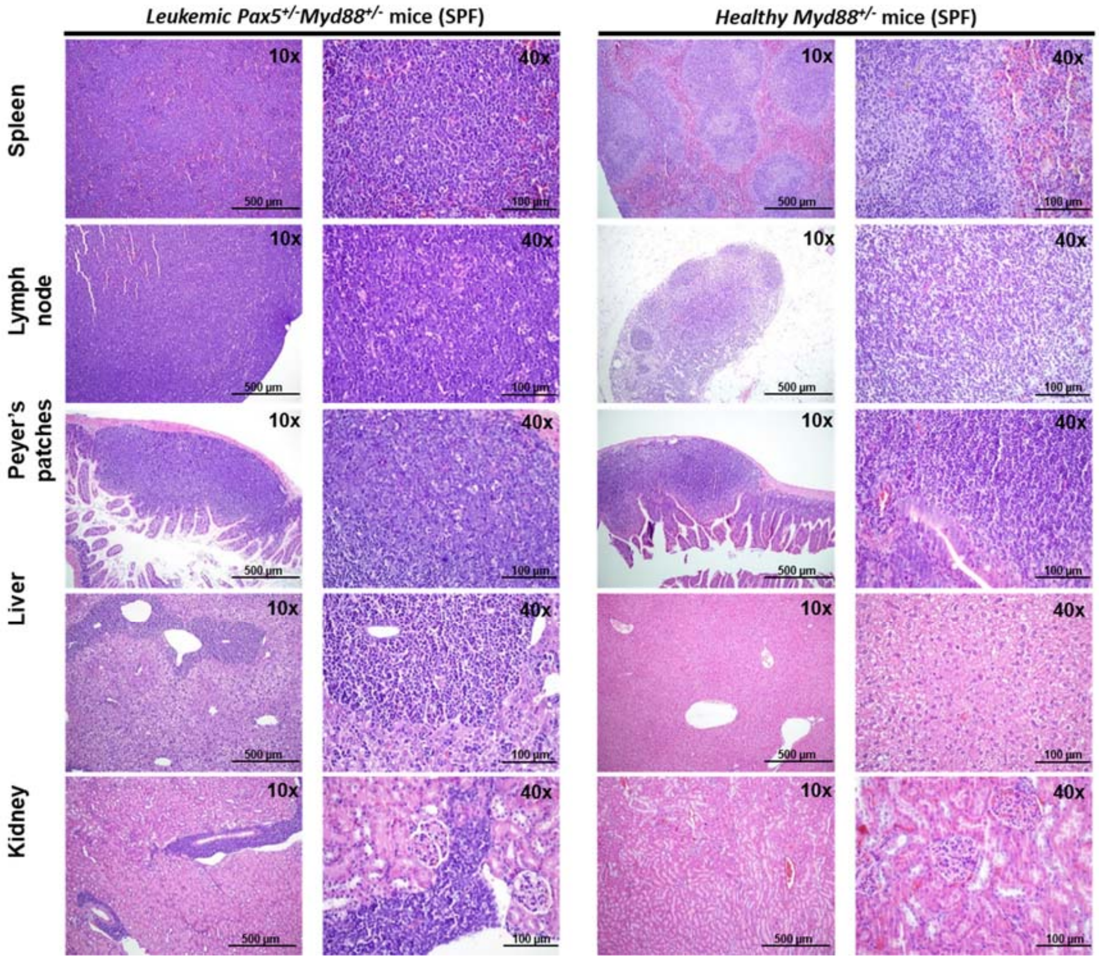

Leukemic Pax5+/-Myd88+/- mice (SPF)
Healthy Myd88+/- mice (SPF)
Spleen
Lymph node
Peyer's patches
Liver
Kidney
10x
40x
500 µm
100 µm

c

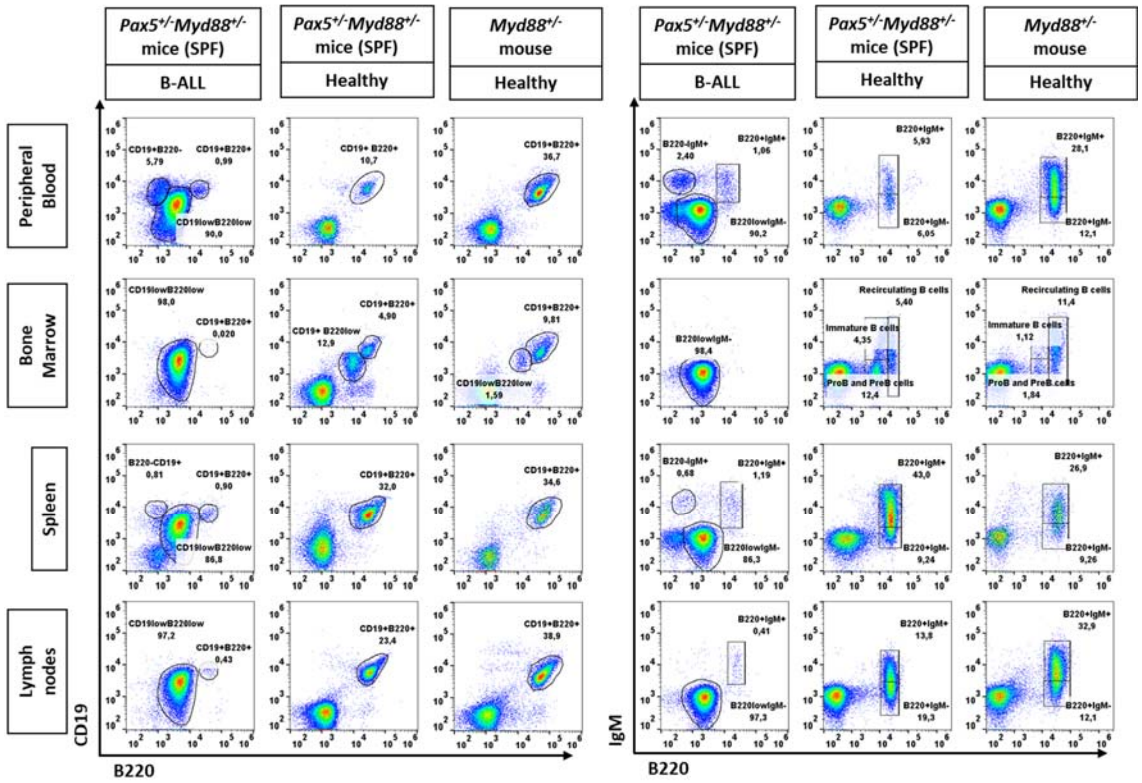

Supplementary Fig. S9: *Pax5*$^{+/-}$*Myd88*$^{+/-}$ mice develop B-ALL without infection exposure. a) B-ALL-specific survival of *Pax5*$^{+/-}$*Myd88*$^{+/-}$ mice (blue line, n=8), *Pax5+/-* mice (red line, n=26), and control wild type (WT) (black line, n=24) mice, all of them housed in an SPF facility (without exposure to common infections). Log-rank (Mantel-Cox) test p-value<0.0001 when comparing *Pax5*$^{+/-}$*Myd88*$^{+/-}$ and *Pax5*$^{+/-}$ mice. Source data are provided as a Source Data file. **b)** Haematoxylin and eosin staining of a tumour-bearing *Pax5*$^{+/-}$*Myd88*$^{+/-}$ mouse unexposed to common infections showing infiltrating blast cells in the spleen, lymph nodes, Peyer's patches, liver, and kidney and compared with a healthy *Myd88*$^{+/-}$ mouse. Loss of normal architecture can be seen due to the infiltrating cells morphologically resembling lymphoblast. Magnification and the corresponding scale bar are indicated in each case. **c)** Flow cytometry representative illustration of the percentage of B cells (CD19^{+}B220^{+} and B220^{+}IgM$^{+/-}$ subsets) in PB, BM, spleen and LN from a diseased *Pax5*$^{+/-}$*Myd88*$^{+/-}$ mouse compared to an age-matched healthy *Pax5*$^{+/-}$*Myd88*$^{+/-}$ mouse, and a healthy *Myd88*$^{+/-}$ mouse.

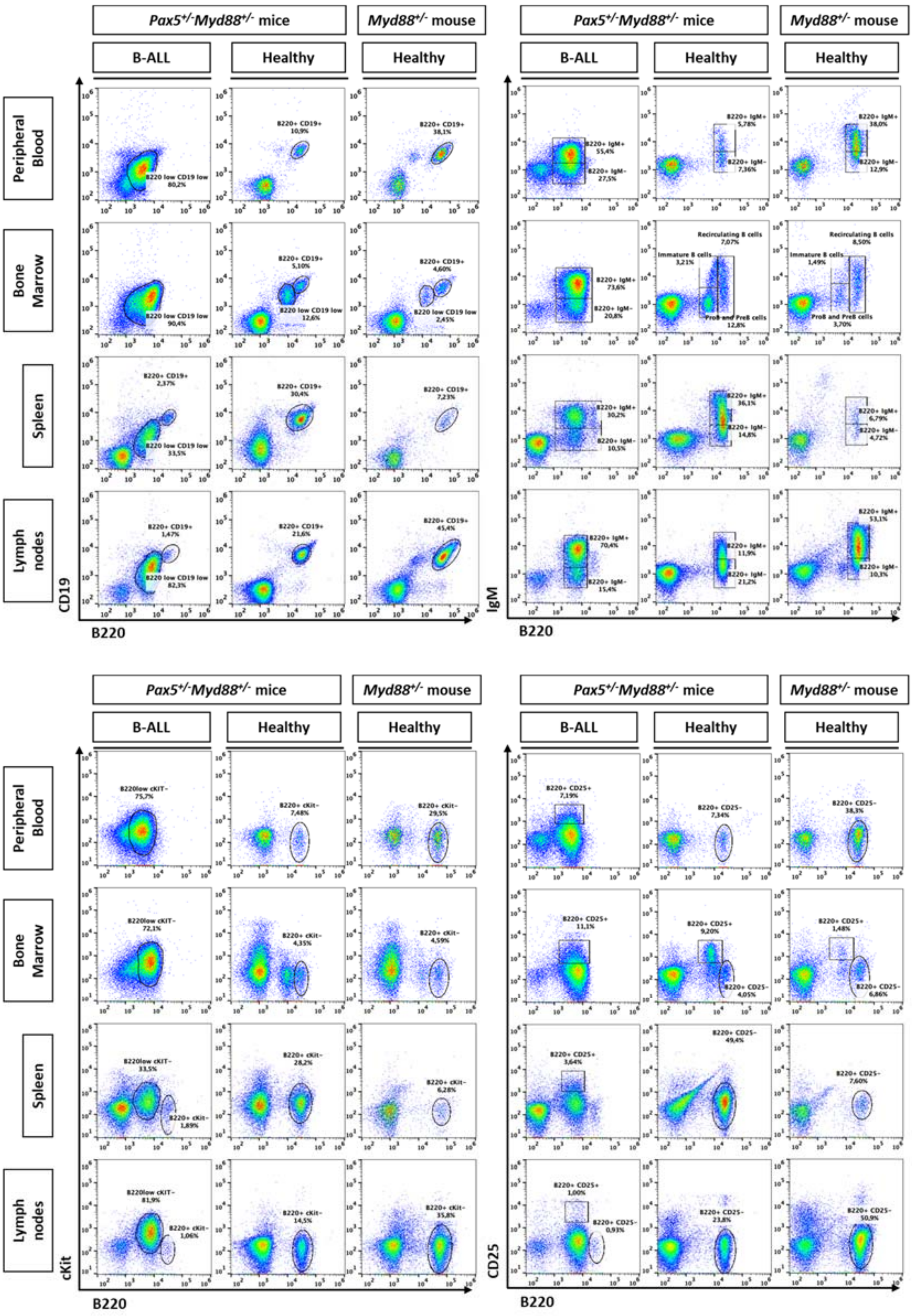

Supplementary Fig. S10: B-ALL in *Pax5$^{+/-}$;Myd88$^{+/-}$* mice. Flow cytometric analysis of hematopoietic subsets in diseased *Pax5$^{+/-}$*;*Myd88$^{+/-}$* mice (X012) a healthy *Myd88$^{+/-}$;Pax5$^{+/-}$* mouse (M706) and a healthy *Myd88$^{+/-}$*mouse (X650). FACS analysis revealed the expected CD19$^{+/-}$B220^{+}IgM^{-}cKit$^{+/-}$CD25$^{+/-}$ cell surface phenotype for B-cell blasts. Representative plots of cell subsets from the peripheral, bone marrow, spleen, and lymph nodes show an accumulation of blast B-cells in leukemic *Pax5$^{+/-}$*;*Myd88$^{+/-}$* mice compared to age-matched healthy *Pax5$^{+/-}$*;*Myd88$^{+/-}$* (M706) mouse.

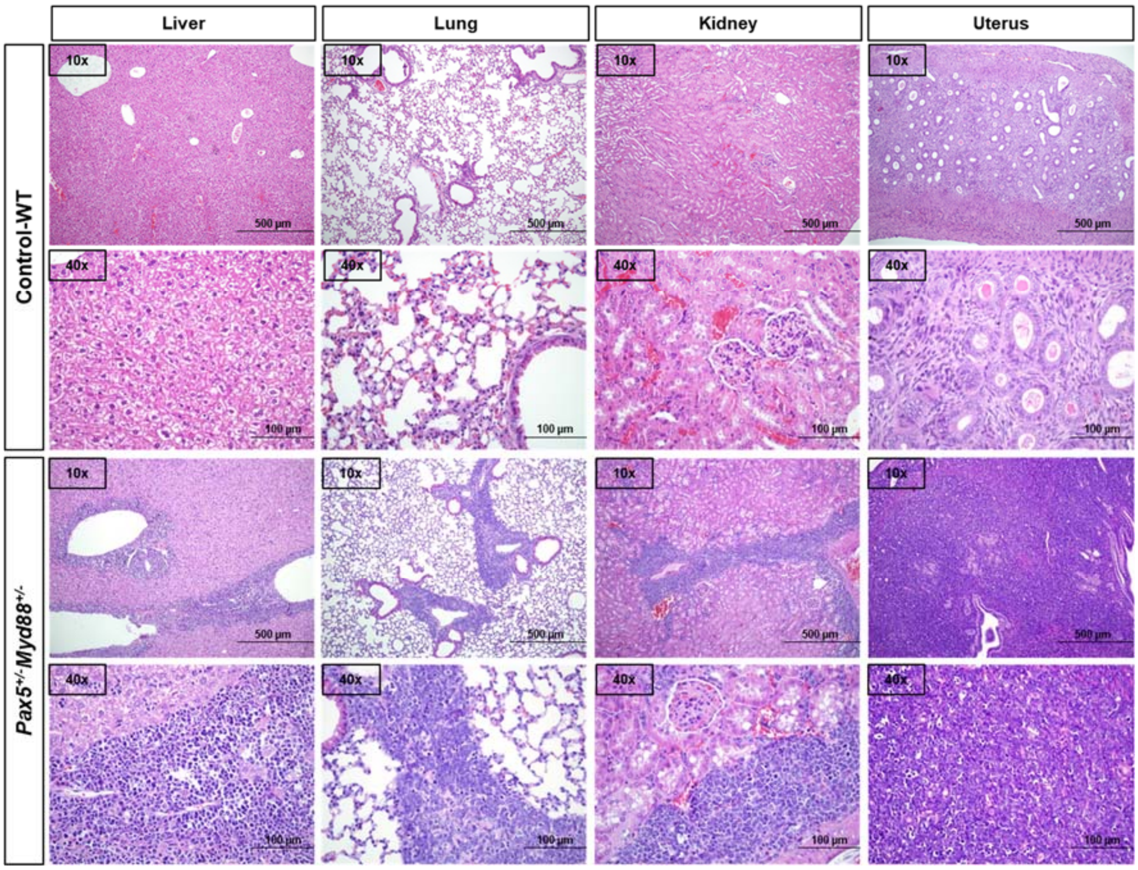

Supplementary Fig. S11: B-ALL infiltration in non-hematological tissues. Haematoxylin and eosin staining of WT mice and tumour-bearing *Pax5*$^{+/-}$;*Myd88*$^{+/-}$ mice (V905 and X012) showing infiltrating blast cells in the liver, lung, kidney, and uterus. Loss of normal architecture due to the accumulation of cells morphologically resembling lymphoblast can be seen in both diseased *Pax5*$^{+/-}$;*Myd88*$^{+/-}$ mice. Magnification and the corresponding scale bar are indicated in each case.

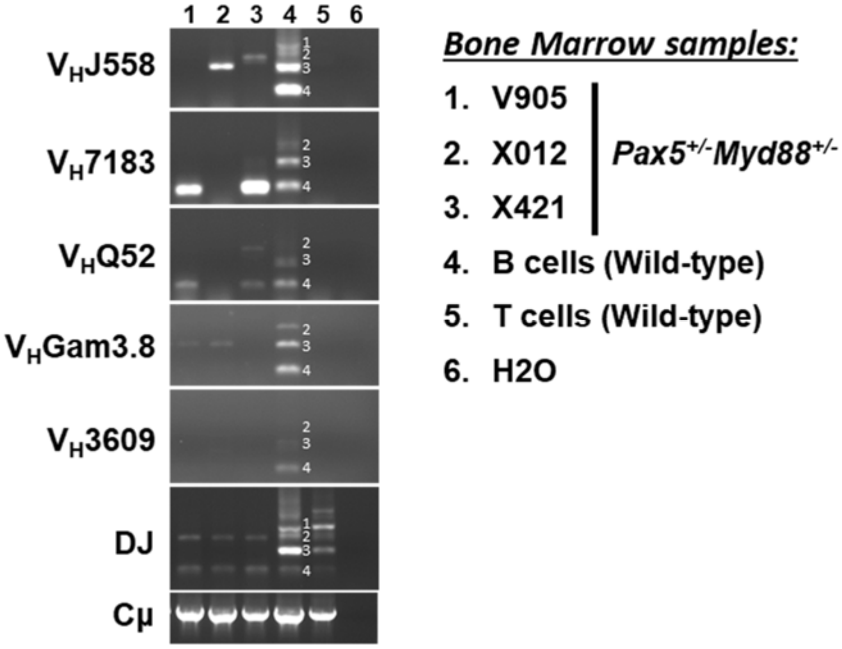

Supplementary Fig. S12: Analysis of BCR clonality of leukemias arising in *Pax5*$^{+/-}$;*Myd88*$^{+/-}$ mice. PCR analysis of BCR gene rearrangements in the bone marrow of diseased mice. Sorted CD19^{+} splenic B cells (B-cells) of healthy mice serve as a control for polyclonal BCR rearrangements. CD8^{+}CD4^{+} T cells from the thymus of healthy mice served as a negative control. Bone marrow leukemic cells show increased clonality within their BCR repertoire (indicated by the code number of each mouse analyzed). Source data are provided as a Source Data file.

a

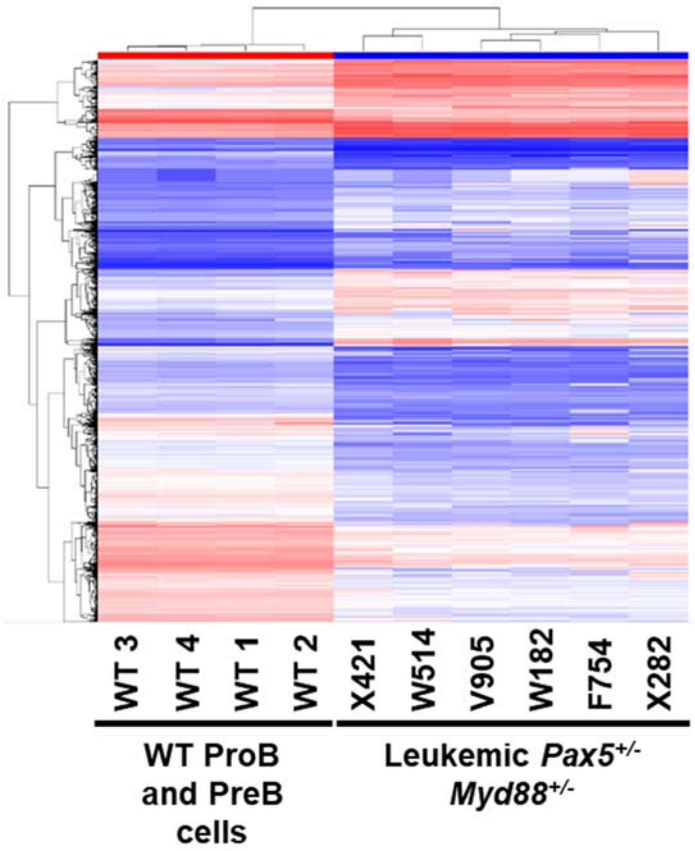

b

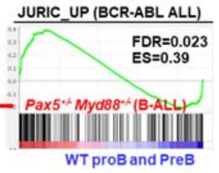

Core enrichment genes

MRC1	mannose receptor, C type 1	PTPLA	protein tyrosine phosphatase-like (proline instead of catalytic arginine), member a
SPARC	secreted acidic cysteine rich glycoprotein	SLC2A5	solute carrier family 2 (facilitated glucose transporter), member 5
ADCY6	adenylate cyclase 6	RUFY3	RUN and FYVE domain containing 3
MLKL	mixed lineage kinase domain-like	ANKRD28	ankyrin repeat domain 28
MYL9	myosin, light polypeptide 9, regulatory	ROBO4	roundabout homolog 4 (Drosophila)
CFH	complement component factor h	PDK4	pyruvate dehydrogenase kinase, isoenzyme 4
MYO1B	myosin IB	RAI14	retinoic acid induced 14
CYYR1	cysteine and tyrosine-rich protein 1	NEDD4	neural precursor cell expressed, developmentally down-regulated 4
GIMAP4	GTPase, IMAP family member 4	CTGF	connective tissue growth factor
PPM1F	protein phosphatase 1F (PP2C domain containing)	RHOB	ras homolog gene family, member B
RALB	v-ral simian leukemia viral oncogene homolog B (ras related)	ICA1	islet cell autoantigen 1
EMP1	epithelial membrane protein 1	TNFRSF1B	tumor necrosis factor receptor superfamily, member 1b
TRIO	triple functional domain (PTPRF interacting)	PKM2	pyruvate kinase, muscle
UACA	uveal autoantigen with coiled-coil domains and ankyrin repeats	TOX	thymocyte selection-associated high mobility group box
OSBPL3	oxysterol binding protein-like 3	CRIM1	cysteine rich transmembrane BMP regulator 1 (chordin like)
CDK6	cyclin-dependent kinase 6	B4GALT1	UDP-Gal:betaGlcNAc beta 1,4- galactosyltransferase, polypeptide 1
ANXA4	annexin A4	SPRED2	sprouty-related, EVH1 domain containing 2
MYLK	myosin, light polypeptide kinase	CRYZ	crystallin, zeta
ATP10A	ATPase, class V, type 10A	GIMAP6	GTPase, IMAP family member 6
HSPD1	heat shock protein 1 (chaperonin)	NADK	NAD kinase
PLAU	plasminogen activator, urokinase	SEMA6A	sema domain, transmembrane domain (TM), and cytoplasmic domain, (semaphorin) 6A
CTBP2	C-terminal binding protein 2	SLC38A1	solute carrier family 38, member 1
PRKCH	protein kinase C, eta	SLC9A3R2	solute carrier family 9 (sodium/hydrogen exchanger), member 3 regulator 2
LOX	lysyl oxidase	EMP2	epithelial membrane protein 2
IL13RA1	interleukin 13 receptor, alpha 1	IL1RAP	interleukin 1 receptor accessory protein
LAMB2	laminin, beta 2	NPDC1	neural proliferation, differentiation and control gene 1
SOCS2	suppressor of cytokine signaling 2	GPA33	glycoprotein A33 (transmembrane)
CASP12	caspase 12	MYO10	myosin X
GAS6	growth arrest specific 6	GBP2	guanylate binding protein 2
COL6A3	collagen, type VI, alpha 3	ETV6	ets variant gene 6 (TEL oncogene)
ODZ4	odd Oz/ten-m homolog 4 (Drosophila)	ERG	avian erythroblastosis virus E-26 (v-ets) oncogene related
POP1	processing of precursor 1, ribonuclease P/MRP family, (S. cerevisiae)	RCN2	reticulocalbin 2
SLC39A10	solute carrier family 39 (zinc transporter), member 10	KCNE3	potassium voltage-gated channel, Isk-related subfamily, gene 3
FAM69B	family with sequence similarity 69, member B	F2R	coagulation factor II (thrombin) receptor
RAB27A	RAB27A, member RAS oncogene family	EAF1	ELL associated factor 1
BACE1	beta-site APP cleaving enzyme 1	GREM1	gremlin 1
DPEP1	dipeptidase 1 (renal)	ZC3H6	zinc finger CCCH type containing 6
CTNND1	catenin (cadherin associated protein), delta 1	GOPC	golgi associated PDZ and coiled-coil motif containing
TNFAIP8L2	tumor necrosis factor, alpha-induced protein 8-like 2	STON2	stonin 2
IRF7	interferon regulatory factor 7	ENG	endoglin
TTN	titin	ITGA5	integrin alpha 5 (fibronectin receptor alpha)
PTPN14	protein tyrosine phosphatase, non-receptor type 14	CTDSPL	CTD (carboxy-terminal domain, RNA polymerase II, polypeptide A) small phosphatase-like
BTNL9	butyrophilin-like 9	NF1	neurofibromatosis 1

c KOHLMANN_UP (BCR-ABL ALL)

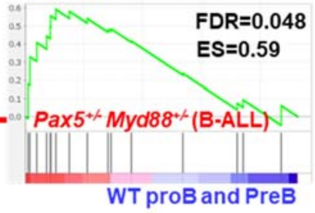

Core enrichment genes

MYO1B	myosin IB
ARHGEF17	Rho guanine nucleotide exchange factor (GEF) 17
APP	amyloid beta (A4) precursor protein
RAI14	retinoic acid induced 14
LGMN	legumain
PXDN	peroxidasin homolog (Drosophila)
EMP2	epithelial membrane protein 2

d LINKA_UP (ETV6-RUNX1 ALL)

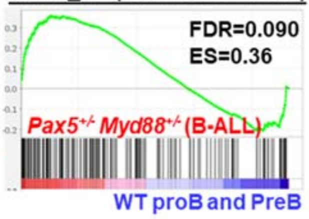

Core enrichment genes

PLCH1	phospholipase C, eta 1
GRB10	growth factor receptor bound protein 10
TNS1	tensin 1
EGFL7	EGF-like domain 7
RGL1	ral guanine nucleotide dissociation stimulator,-like 1
FLT3	FMS-like tyrosine kinase 3
TGM2	transglutaminase 2, C polypeptide
TFPI	tissue factor pathway inhibitor
ARHGEF17	Rho guanine nucleotide exchange factor (GEF) 17
VPREB1	pre-B lymphocyte gene 1
TSPAN7	tetraspanin 7
GUCY1A3	guanylate cyclase 1, soluble, alpha 3
MME	membrane metallo endopeptidase
ANGPTL4	angiopoietin-like 4
MYH10	myosin, heavy polypeptide 10, non-muscle
SOCS2	suppressor of cytokine signaling 2
ODZ4	odd Oz/ten-m homolog 4 (Drosophila)
MYO18B	myosin XVIIIb
SMAD1	MAD homolog 1 (Drosophila)
FAM69B	family with sequence similarity 69, member B
DYRK3	dual-specificity tyrosine-(Y)-phosphorylation regulated kinase 3
DPEP1	dipeptidase 1 (renal)
GTF2IRD1	general transcription factor II I repeat domain-containing 1
TMED6	transmembrane emp24 protein transport domain containing 6
PTPLA	protein tyrosine phosphatase-like (proline instead of catalytic arginine), member a
CLEC14A	C-type lectin domain family 14, member a
TNFRSF21	tumor necrosis factor receptor superfamily, member 21
LALBA	lactalbumin, alpha
PXDN	peroxidasin homolog (Drosophila)
DBN1	drebrin 1
PLXNB1	plexin B1
FSCN1	fascin homolog 1, actin bundling protein (Strongylocentrotus purpuratus)
SHISA2	shisa homolog 2 (Xenopus laevis)
NPR1	natriuretic peptide receptor 1
ECE2	endothelin converting enzyme 2
PCDHB11	protocadherin beta 11

Supplementary Fig. S13: Gene expression analysis of leukemic *Pax5*$^{+/-}$;*Myd88*$^{+/-}$ mice. a) Unsupervised heatmap showing the differentially expressed genes (n=2463) between bone marrow leukemic cells from *Pax5*$^{+/-}$;*Myd88*$^{+/-}$ mice (n=6) and proB and preB cells (bone marrow B220^{low} IgM^{+}cells) from control wild type (WT) mice (n=4). The significance analysis of microarrays was defined by a p-value<0.05 and fold change: FC<-2 or FC>2. **b-d)** GSEA showing that leukemic Pax5$^{+/-}$;Myd88$^{+/-}$ cells from diseased mice present similar profiles for genesets previously identified in human BCR-ABL+ and ETV6-RUNX1+ B-ALL samples[1-3]. The list of genes in the core enrichment is indicated in each case.

a

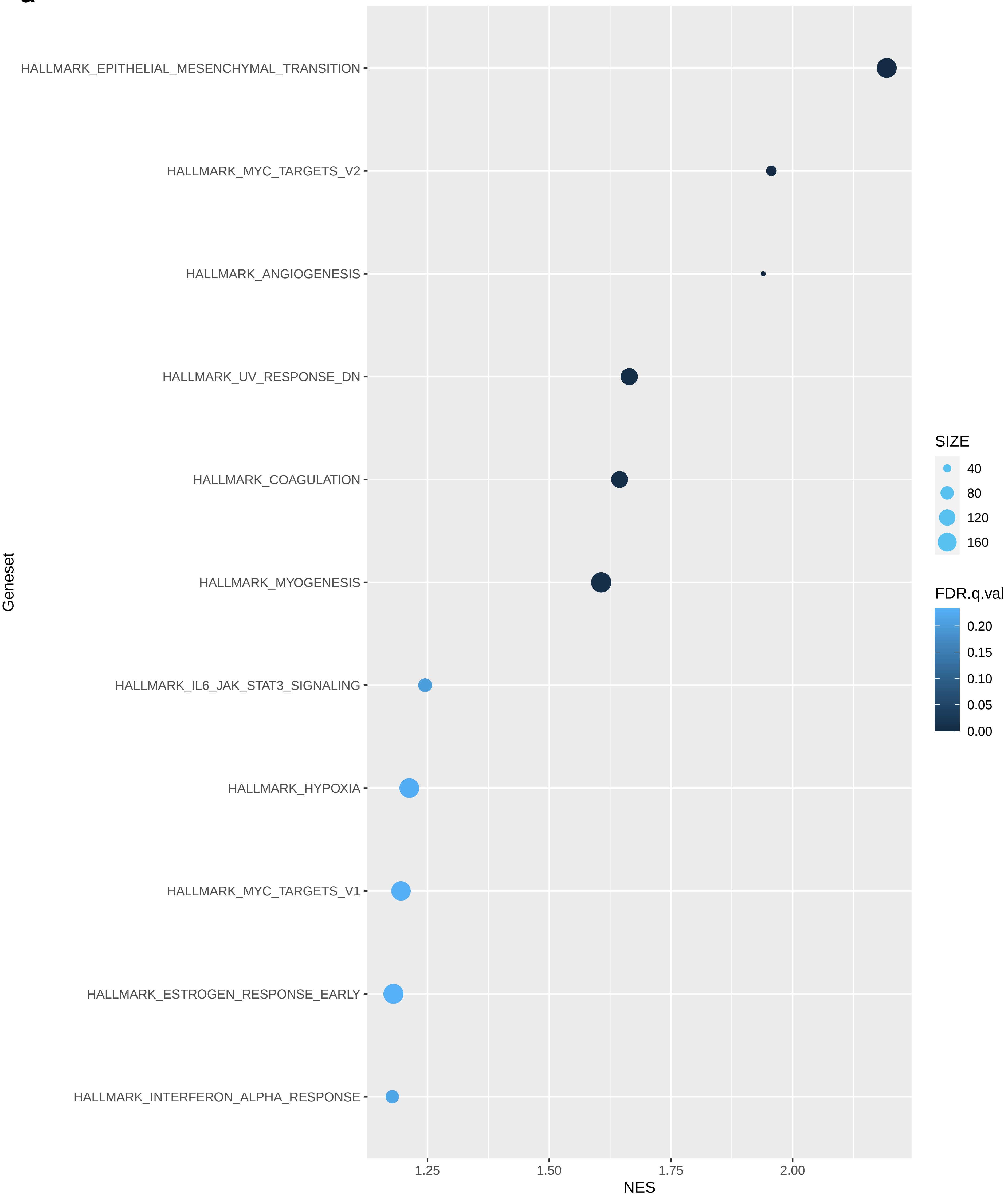

HALLMARK_EPITHELIAL_MESENCHYMAL_TRANSITION
HALLMARK_MYC_TARGETS_V2
HALLMARK_ANGIOGENESIS
HALLMARK_UV_RESPONSE_DN
HALLMARK_COAGULATION
HALLMARK_MYOGENESIS
HALLMARK_IL6_JAK_STAT3_SIGNALING
HALLMARK_HYPOXIA
HALLMARK_MYC_TARGETS_V1
HALLMARK_ESTROGEN_RESPONSE_EARLY
HALLMARK_INTERFERON_ALPHA_RESPONSE
Geneset
1.25
1.50
1.75
2.00
NES
SIZE
40
80
120
160
FDR.q.val
0.20
0.15
0.10
0.05
0.00

b

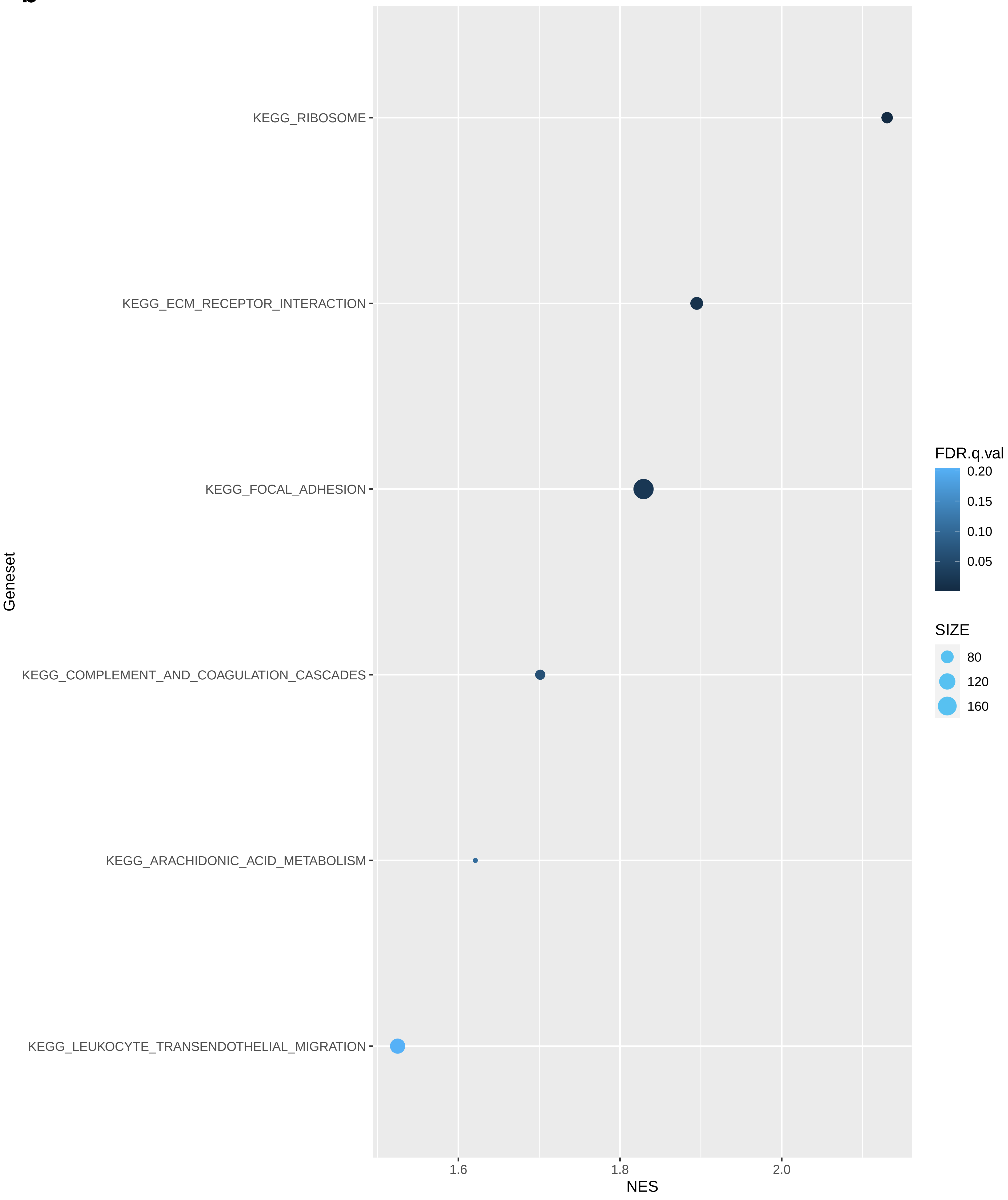
KEGG_RIBOSOME
KEGG_ECM_RECEPTOR_INTERACTION
KEGG_FOCAL_ADHESION
KEGG_COMPLEMENT_AND_COAGULATION_CASCADES
KEGG_ARACHIDONIC_ACID_METABOLISM
KEGG_LEUKOCYTE_TRANSENDOTHELIAL_MIGRATION
Geneset
1.6
1.8
2.0
NES
FDR.q.val
0.20
0.15
0.10
0.05
SIZE
80
120
160

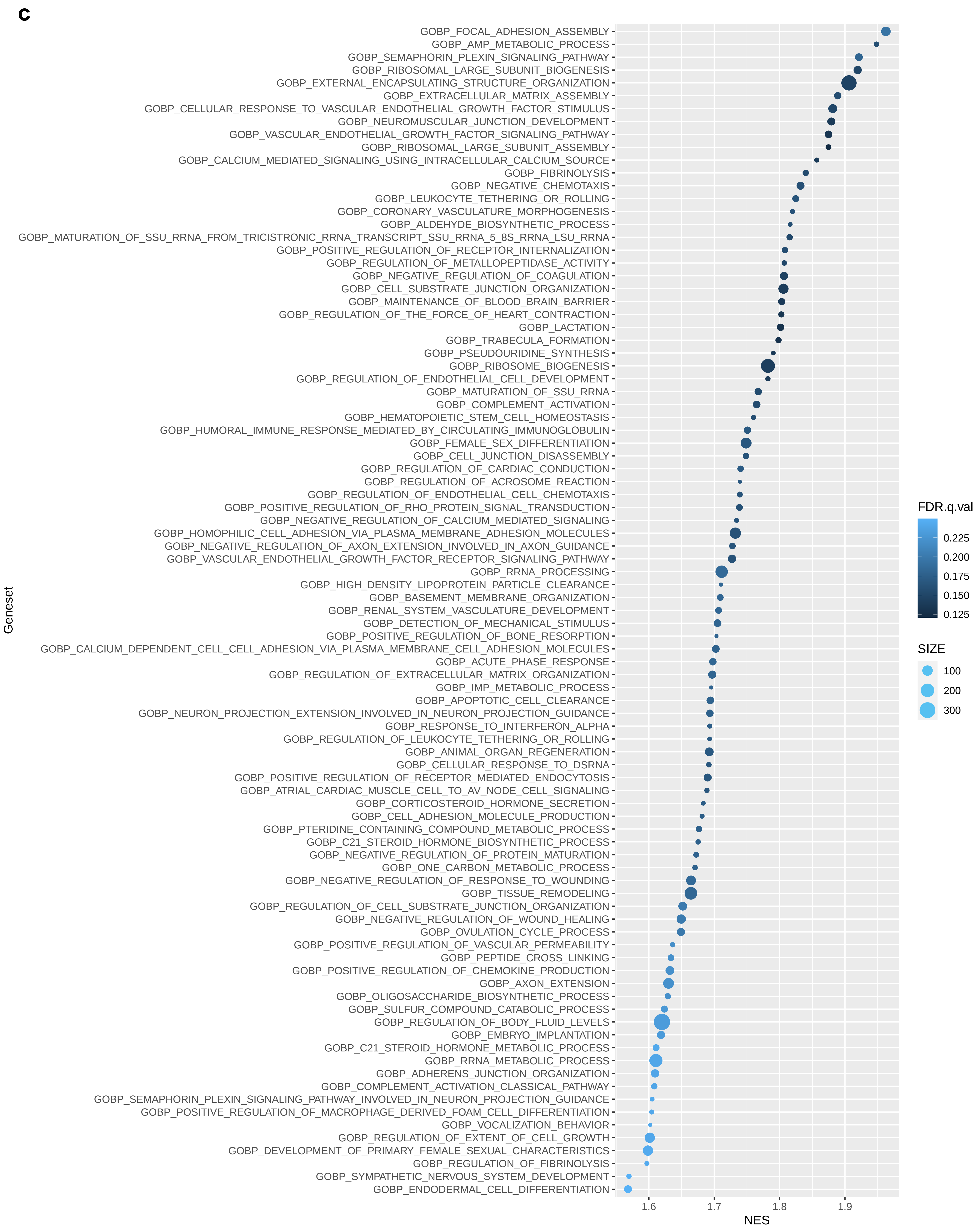
c
GOBP_FOCAL_ADHESION_ASSEMBLY
GOBP_AMP_METABOLIC_PROCESS
GOBP_SEMAPHORIN_PLEXIN_SIGNALING_PATHWAY
GOBP_RIBOSOMAL_LARGE_SUBUNIT_BIOGENESIS
GOBP_EXTERNAL_ENCAPSULATING_STRUCTURE_ORGANIZATION
GOBP_EXTRACELLULAR_MATRIX_ASSEMBLY
GOBP_CELLULAR_RESPONSE_TO_VASCULAR_ENDOTHELIAL_GROWTH_FACTOR_STIMULUS
GOBP_NEUROMUSCULAR_JUNCTION_DEVELOPMENT
GOBP_VASCULAR_ENDOTHELIAL_GROWTH_FACTOR_SIGNALING_PATHWAY
GOBP_RIBOSOMAL_LARGE_SUBUNIT_ASSEMBLY
GOBP_CALCIUM_MEDIATED_SIGNALING_USING_INTRACELLULAR_CALCIUM_SOURCE
GOBP_FIBRINOLYSIS
GOBP_NEGATIVE_CHEMOTAXIS
GOBP_LEUKOCYTE_TETHERING_OR_ROLLING
GOBP_CORONARY_VASCULATURE_MORPHOGENESIS
GOBP_ALDEHYDE_BIOSYNTHETIC_PROCESS
GOBP_MATURATION_OF_SSU_RRNA_FROM_TRICISTRONIC_RRNA_TRANSCRIPT_SSU_RRNA_5_8S_RRNA_LSU_RRNA
GOBP_POSITIVE_REGULATION_OF_RECEPTOR_INTERNALIZATION
GOBP_REGULATION_OF_METALLOPEPTIDASE_ACTIVITY
GOBP_NEGATIVE_REGULATION_OF_COAGULATION
GOBP_CELL_SUBSTRATE_JUNCTION_ORGANIZATION
GOBP_MAINTENANCE_OF_BLOOD_BRAIN_BARRIER
GOBP_REGULATION_OF_THE_FORCE_OF_HEART_CONTRACTION
GOBP_LACTATION
GOBP_TRABECULA_FORMATION
GOBP_PSEUDOURIDINE_SYNTHESIS
GOBP_RIBOSOME_BIOGENESIS
GOBP_REGULATION_OF_ENDOTHELIAL_CELL_DEVELOPMENT
GOBP_MATURATION_OF_SSU_RRNA
GOBP_COMPLEMENT_ACTIVATION
GOBP_HEMATOPOIETIC_STEM_CELL_HOMEOSTASIS
GOBP_HUMORAL_IMMUNE_RESPONSE_MEDIATED_BY_CIRCULATING_IMMUNOGLOBULIN
GOBP_FEMALE_SEX_DIFFERENTIATION
GOBP_CELL_JUNCTION_DISASSEMBLY
GOBP_REGULATION_OF_CARDIAC_CONDUCTION
GOBP_REGULATION_OF_ACROSOME_REACTION
GOBP_REGULATION_OF_ENDOTHELIAL_CELL_CHEMOTAXIS
GOBP_POSITIVE_REGULATION_OF_RHO_PROTEIN_SIGNAL_TRANSDUCTION
GOBP_NEGATIVE_REGULATION_OF_CALCIUM_MEDIATED_SIGNALING
GOBP_HOMOPHILIC_CELL_ADHESION_VIA_PLASMA_MEMBRANE_ADHESION_MOLECULES
GOBP_NEGATIVE_REGULATION_OF_AXON_EXTENSION_INVOLVED_IN_AXON_GUIDANCE
GOBP_VASCULAR_ENDOTHELIAL_GROWTH_FACTOR_RECEPTOR_SIGNALING_PATHWAY
GOBP_RRNA_PROCESSING
GOBP_HIGH_DENSITY_LIPOPROTEIN_PARTICLE_CLEARANCE
GOBP_BASEMENT_MEMBRANE_ORGANIZATION
GOBP_RENAL_SYSTEM_VASCULATURE_DEVELOPMENT
GOBP_DETECTION_OF_MECHANICAL_STIMULUS
GOBP_POSITIVE_REGULATION_OF_BONE_RESORPTION
GOBP_CALCIUM_DEPENDENT_CELL_CELL_ADHESION_VIA_PLASMA_MEMBRANE_CELL_ADHESION_MOLECULES
GOBP_ACUTE_PHASE_RESPONSE
GOBP_REGULATION_OF_EXTRACELLULAR_MATRIX_ORGANIZATION
GOBP_IMP_METABOLIC_PROCESS
GOBP_APOPTOTIC_CELL_CLEARANCE
GOBP_NEURON_PROJECTION_EXTENSION_INVOLVED_IN_NEURON_PROJECTION_GUIDANCE
GOBP_RESPONSE_TO_INTERFERON_ALPHA
GOBP_REGULATION_OF_LEUKOCYTE_TETHERING_OR_ROLLING
GOBP_ANIMAL_ORGAN_REGENERATION
GOBP_CELLULAR_RESPONSE_TO_DSRNA
GOBP_POSITIVE_REGULATION_OF_RECEPTOR_MEDIATED_ENDOCYTOSIS
GOBP_ATRIAL_CARDIAC_MUSCLE_CELL_TO_AV_NODE_CELL_SIGNALING
GOBP_CORTICOSTEROID_HORMONE_SECRETION
GOBP_CELL_ADHESION_MOLECULE_PRODUCTION
GOBP_PTERIDINE_CONTAINING_COMPOUND_METABOLIC_PROCESS
GOBP_C21_STEROID_HORMONE_BIOSYNTHETIC_PROCESS
GOBP_NEGATIVE_REGULATION_OF_PROTEIN_MATURATION
GOBP_ONE_CARBON_METABOLIC_PROCESS
GOBP_NEGATIVE_REGULATION_OF_RESPONSE_TO_WOUNDING
GOBP_TISSUE_REMODELING
GOBP_REGULATION_OF_CELL_SUBSTRATE_JUNCTION_ORGANIZATION
GOBP_NEGATIVE_REGULATION_OF_WOUND_HEALING
GOBP_OVULATION_CYCLE_PROCESS
GOBP_POSITIVE_REGULATION_OF_VASCULAR_PERMEABILITY
GOBP_PEPTIDE_CROSS_LINKING
GOBP_POSITIVE_REGULATION_OF_CHEMOKINE_PRODUCTION
GOBP_AXON_EXTENSION
GOBP_OLIGOSACCHARIDE_BIOSYNTHETIC_PROCESS
GOBP_SULFUR_COMPOUND_CATABOLIC_PROCESS
GOBP_REGULATION_OF_BODY_FLUID_LEVELS
GOBP_EMBRYO_IMPLANTATION
GOBP_C21_STEROID_HORMONE_METABOLIC_PROCESS
GOBP_RRNA_METABOLIC_PROCESS
GOBP_ADHERENS_JUNCTION_ORGANIZATION
GOBP_COMPLEMENT_ACTIVATION_CLASSICAL_PATHWAY
GOBP_SEMAPHORIN_PLEXIN_SIGNALING_PATHWAY_INVOLVED_IN_NEURON_PROJECTION_GUIDANCE
GOBP_POSITIVE_REGULATION_OF_MACROPHAGE_DERIVED_FOAM_CELL_DIFFERENTIATION
GOBP_VOCALIZATION_BEHAVIOR
GOBP_REGULATION_OF_EXTENT_OF_CELL_GROWTH
GOBP_DEVELOPMENT_OF_PRIMARY_FEMALE_SEXUAL_CHARACTERISTICS
GOBP_REGULATION_OF_FIBRINOLYSIS
GOBP_SYMPATHETIC_NERVOUS_SYSTEM_DEVELOPMENT
GOBP_ENDODERMAL_CELL_DIFFERENTIATION
Geneset
1.6
1.7
1.8
1.9
NES
FDR.q.val
0.225
0.200
0.175
0.150
0.125
SIZE
100
200
300

Supplementary Fig. S14: Gene expression enrichment analysis of leukemic Pax5$^{+/-}$;Myd88$^{+/-}$ mice. Gene Set Enrichment Analyses were performed using the gene sets from hallmark collection database **a)**, canonical pathways gene sets derived from the KEGG pathway database **b),** and gene sets derived from the GO Biological Process Ontology **c)** from MSigDB[4-6]. This analysis identifies significant enrichment of several pathways in Myd88$^{+/-}$Pax5$^{+/-}$ (B-ALL) plotted on the y-axis in comparison to proB and preB cells (bone marrow B220^{low} IgM^{+}cells) from control wild-type (WT) mice. On the x-axis, the NES (Normalized Enrichment Score) value is represented for each gene set. The corresponding FDR (False Discovery Rate) value is represented in a blue color scale and the dot size depicts the gene count. Source data are provided as a Source Data file.

a

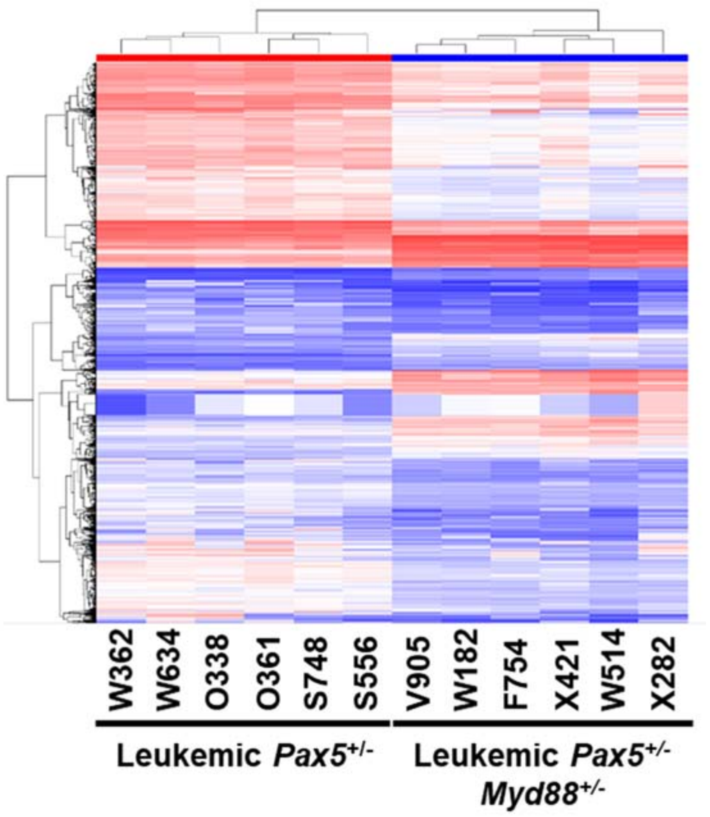
W362
W634
O338
O361
S748
S556
V905
W182
F754
X421
W514
X282
Leukemic Pax5+/-
Leukemic Pax5+/- Myd88+/-

b

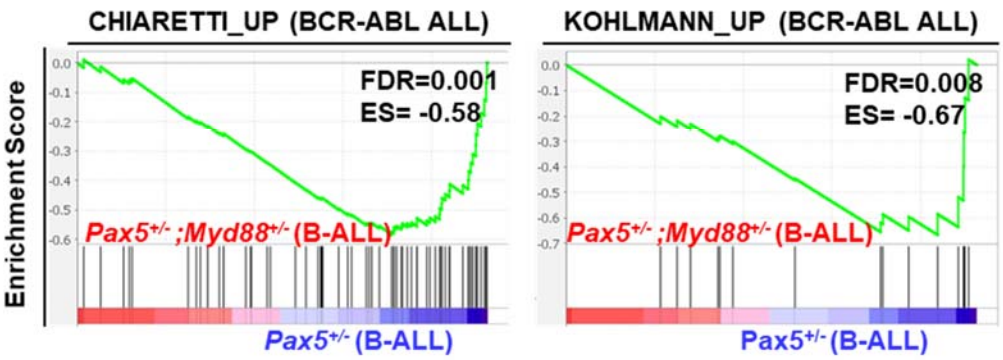
CHIARETTI_UP (BCR-ABL ALL)
KOHLMANN_UP (BCR-ABL ALL)
Enrichment Score
FDR=0.001
ES= -0.58
FDR=0.008
ES= -0.67
Pax5+/- ;Myd88+/- (B-ALL)
Pax5+/- ;Myd88+/- (B-ALL)
Pax5+/- (B-ALL)
Pax5+/- (B-ALL)

c

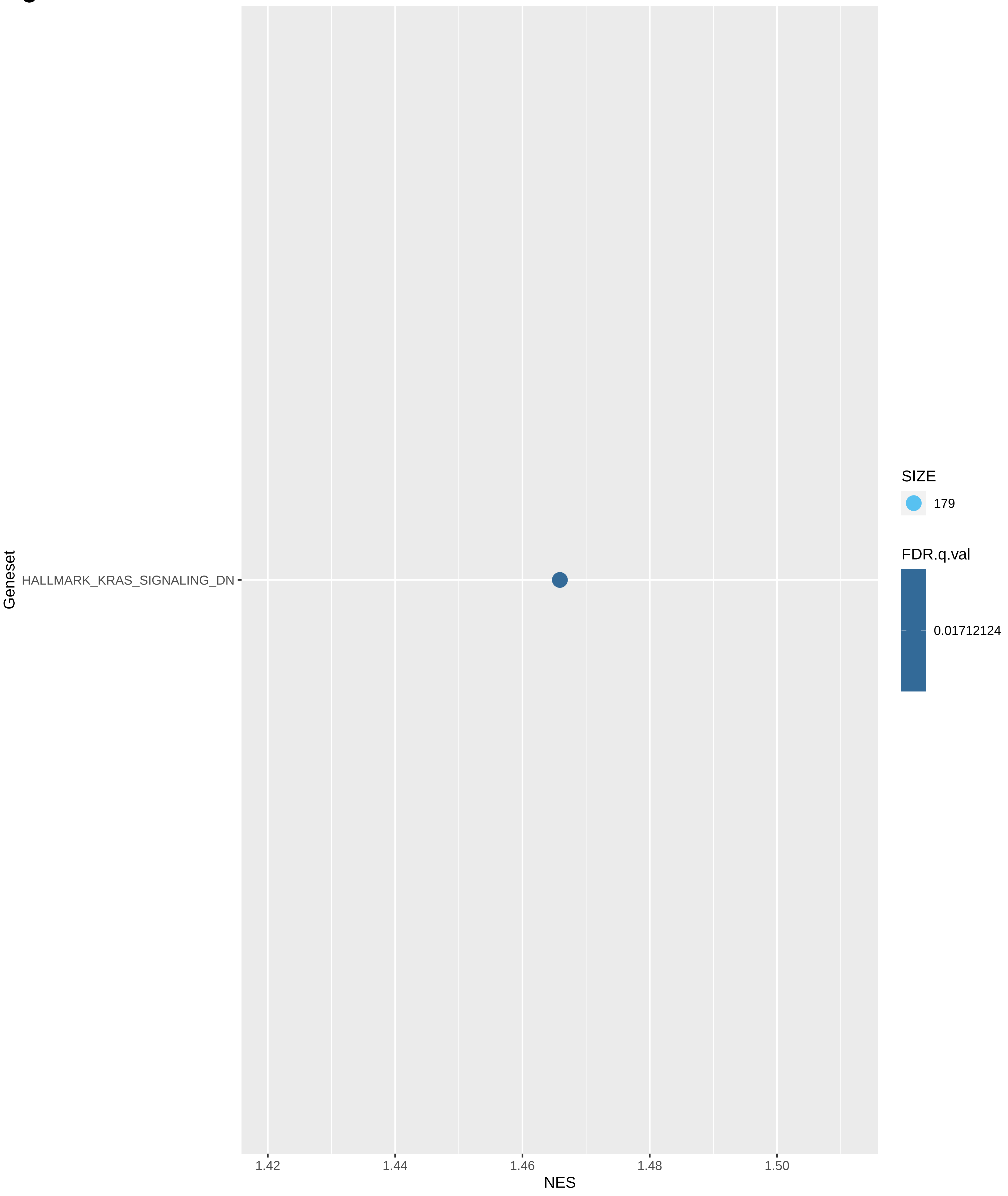
SIZE
179
FDR.q.val
0.01712124
Geneset
HALLMARK_KRAS_SIGNALING_DN
1.42
1.44
1.46
1.48
1.50
NES

d

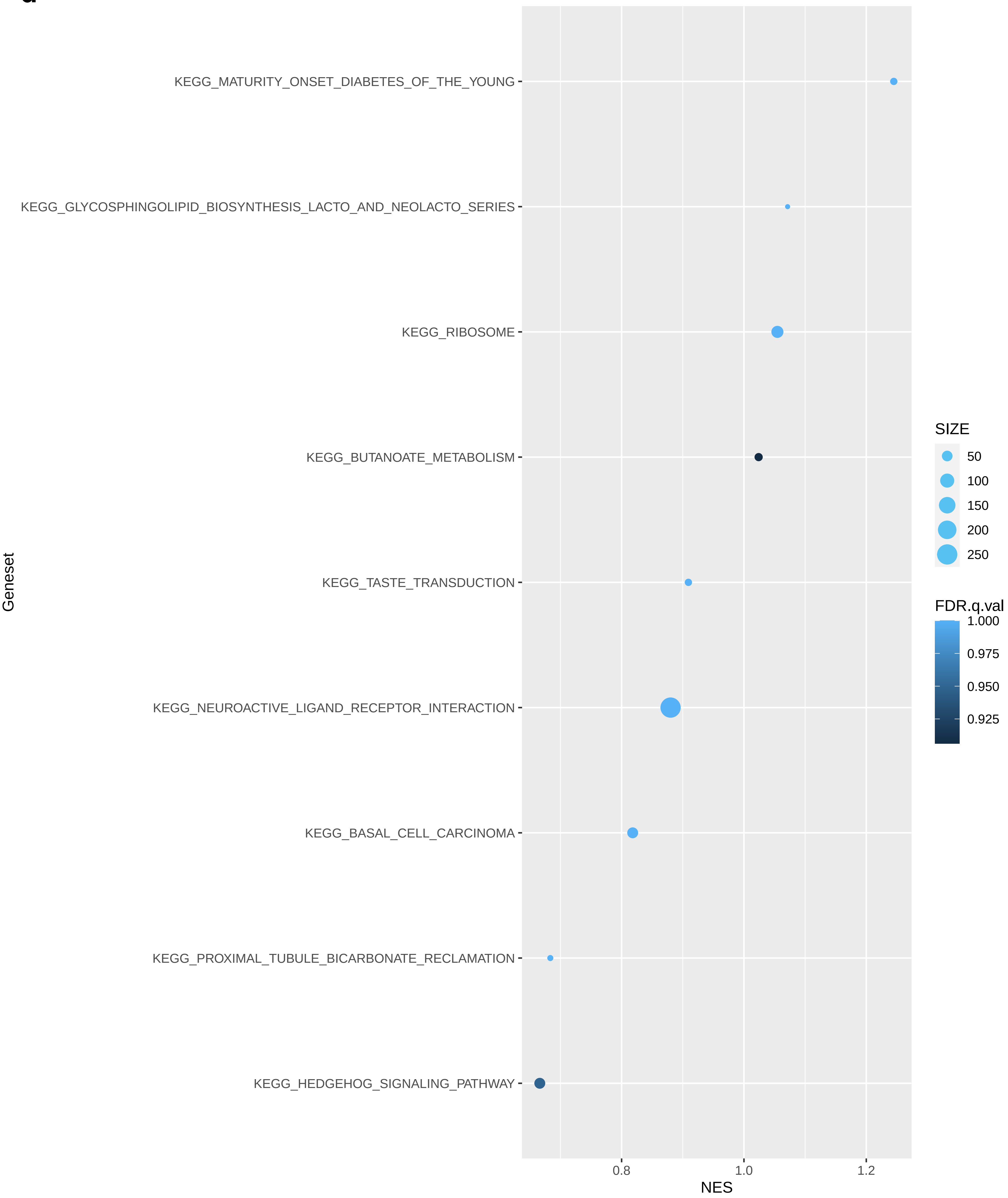

KEGG_MATURITY_ONSET_DIABETES_OF_THE_YOUNG
KEGG_GLYCOSPHINGOLIPID_BIOSYNTHESIS_LACTO_AND_NEOLACTO_SERIES
KEGG_RIBOSOME
KEGG_BUTANOATE_METABOLISM
KEGG_TASTE_TRANSDUCTION
KEGG_NEUROACTIVE_LIGAND_RECEPTOR_INTERACTION
KEGG_BASAL_CELL_CARCINOMA
KEGG_PROXIMAL_TUBULE_BICARBONATE_RECLAMATION
KEGG_HEDGEHOG_SIGNALING_PATHWAY
Geneset
0.8
1.0
1.2
NES
SIZE
50
100
150
200
250
FDR.q.val
1.000
0.975
0.950
0.925

e

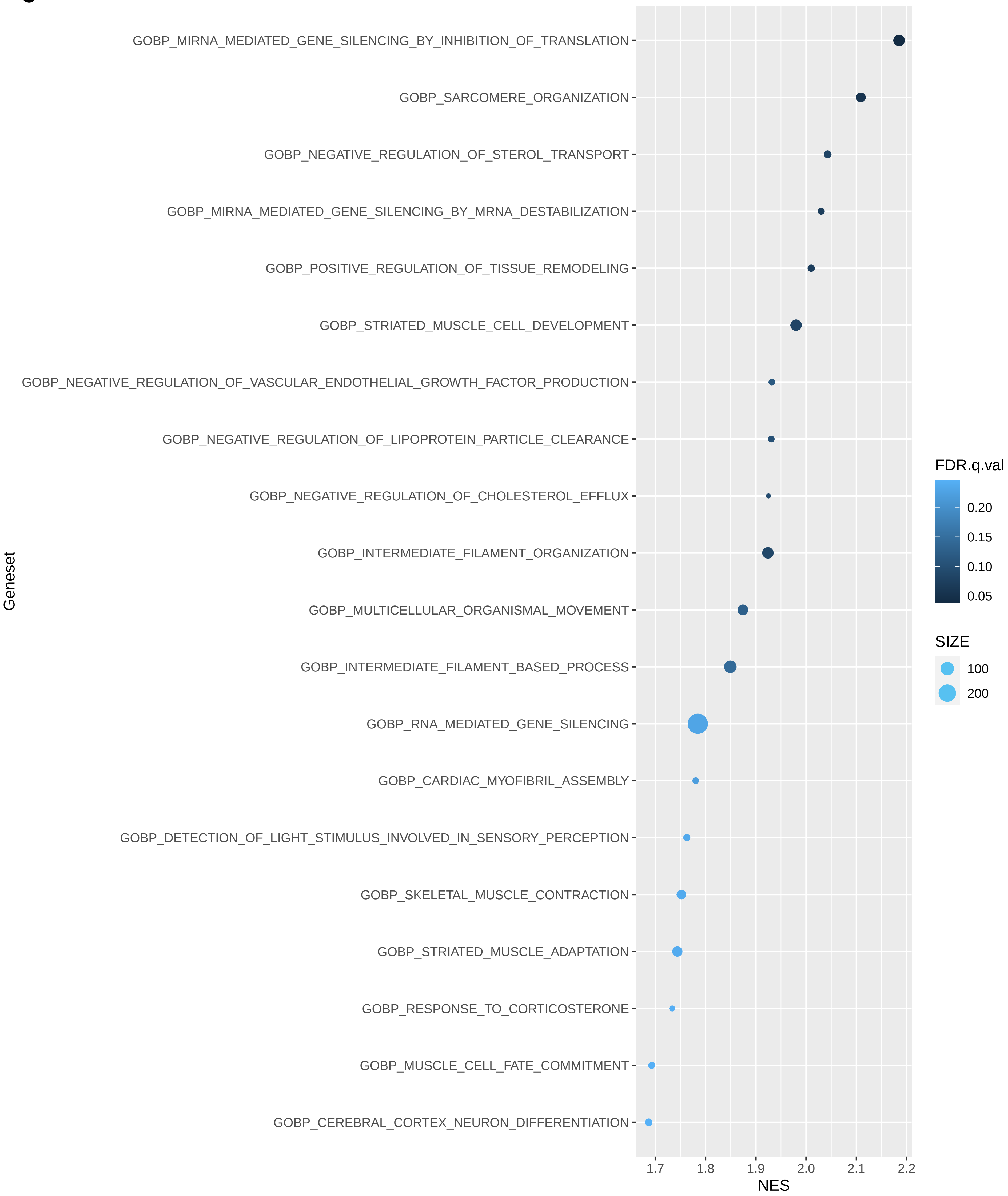
GOBP_MIRNA_MEDIATED_GENE_SILENCING_BY_INHIBITION_OF_TRANSLATION
GOBP_SARCOMERE_ORGANIZATION
GOBP_NEGATIVE_REGULATION_OF_STEROL_TRANSPORT
GOBP_MIRNA_MEDIATED_GENE_SILENCING_BY_MRNA_DESTABILIZATION
GOBP_POSITIVE_REGULATION_OF_TISSUE_REMODELING
GOBP_STRIATED_MUSCLE_CELL_DEVELOPMENT
GOBP_NEGATIVE_REGULATION_OF_VASCULAR_ENDOTHELIAL_GROWTH_FACTOR_PRODUCTION
GOBP_NEGATIVE_REGULATION_OF_LIPOPROTEIN_PARTICLE_CLEARANCE
GOBP_NEGATIVE_REGULATION_OF_CHOLESTEROL_EFFLUX
GOBP_INTERMEDIATE_FILAMENT_ORGANIZATION
GOBP_MULTICELLULAR_ORGANISMAL_MOVEMENT
GOBP_INTERMEDIATE_FILAMENT_BASED_PROCESS
GOBP_RNA_MEDIATED_GENE_SILENCING
GOBP_CARDIAC_MYOFIBRIL_ASSEMBLY
GOBP_DETECTION_OF_LIGHT_STIMULUS_INVOLVED_IN_SENSORY_PERCEPTION
GOBP_SKELETAL_MUSCLE_CONTRACTION
GOBP_STRIATED_MUSCLE_ADAPTATION
GOBP_RESPONSE_TO_CORTICOSTERONE
GOBP_MUSCLE_CELL_FATE_COMMITMENT
GOBP_CEREBRAL_CORTEX_NEURON_DIFFERENTIATION
Geneset
1.7
1.8
1.9
2.0
2.1
2.2
NES
FDR.q.val
0.20
0.15
0.10
0.05
SIZE
100
200

Supplementary Fig. S15: Gene expression analysis of leukemic *Pax5*$^{+/-}$;*Myd88*$^{+/-}$ mice compared with leukemic *Pax5*$^{+/-}$ mice. a) Unsupervised heatmap showing the differentially expressed genes (n=1385) between leukemic cells from *Pax5*$^{+/-}$;*Myd88*$^{+/-}$ mice (n=6) and leukemic cells from *Pax5*$^{+/-}$ mice (n=6). The significance analysis of microarrays was defined by a p-value<0.05 and fold change: FC<-2 or FC>2. **b**) GSEA showing that leukemic *Pax5*$^{+/-}$ cells from diseased mice are upregulated in signatures for human B-ALL **c-d)** Enrichment analysis of leukemic cells from Pax5$^{+/-}$;Myd88$^{+/-}$ mice compared with leukemic *Pax5*$^{+/-}$ mice. Gene Set Enrichment Analyses were performed using the gene sets from the hallmark collection database **c**), canonical pathways gene sets derived from the KEGG pathway database **d**) and gene sets derived from the GO Biological Process Ontology **e**) from MSigDB[4-6]. This analysis identifies enriched pathways in Myd88$^{+/-}$Pax5$^{+/-}$ (B-ALL) plotted on the y-axis. On the x-axis, the NES (Normalized Enrichment Score) value is represented for each gene set. The corresponding FDR (False Discovery Rate) value is represented in a blue color scale and the dot size depicts the gene count. Source data are provided as a Source Data file.

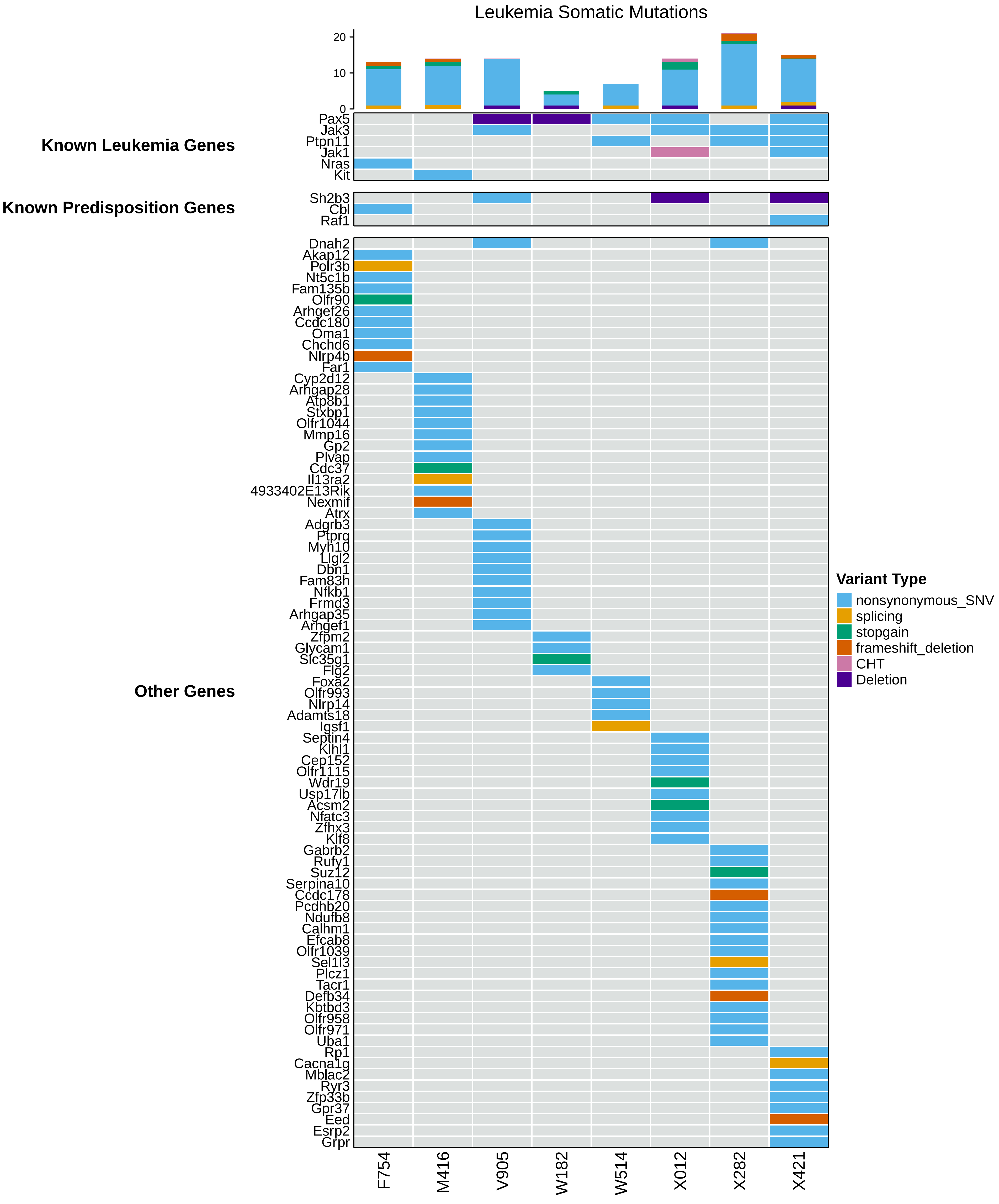
Leukemia Somatic Mutations
0
10
20
Known Leukemia Genes
Pax5
Jak3
Ptpn11
Jak1
Nras
Kit
Known Predisposition Genes
Sh2b3
Cbl
Raf1
Other Genes
Dnah2
Akap12
Polr3b
Nt5c1b
Fam135b
Olfr90
Arhgef26
Ccdc180
Oma1
Chchd6
Nlrp4b
Far1
Cyp2d12
Arhgap28
Atp8b1
Stxbp1
Olfr1044
Mmp16
Gp2
Plvap
Cdc37
Il13ra2
4933402E13Rik
Nexmif
Atrx
Adgrb3
Ptprq
Myh10
Llgl2
Dbn1
Fam83h
Nfkb1
Frmd3
Arhgap35
Arhgef1
Zfpm2
Glycam1
Slc35g1
Flg2
Foxa2
Olfr993
Nlrp14
Adamts18
Igsf1
Septin4
Klhl1
Cep152
Olfr1115
Wdr19
Usp17lb
Acsm2
Nfatc3
Zfhx3
Klf8
Gabrb2
Rufy1
Suz12
Serpina10
Ccdc178
Pcdhb20
Ndufb8
Calhm1
Efcab8
Olfr1039
Sel1l3
Plcz1
Tacr1
Defb34
Kbtbd3
Olfr958
Olfr971
Uba1
Rp1
Cacna1g
Mblac2
Ryr3
Zfp33b
Gpr37
Eed
Esrp2
Grpr
F754
M416
V905
W182
W514
X012
X282
X421
Variant Type
nonsynonymous_SNV
splicing
stopgain
frameshift_deletion
CHT
Deletion

Supplementary Fig. S16: Whole Genome Sequencing in leukemic *Pax5*$^{+/-}$;*Myd88*$^{+/-}$ mice. Oncoprint of genes with somatic mutations found in 8 leukemia samples from *Pax5*$^{+/-}$;*Myd88*$^{+/-}$ mice. Somatic alterations are clustered by gene. Tumor DNA was derived from whole leukemic bone marrow or lymph nodes, while tail DNA of the respective mouse was used as reference germline material.

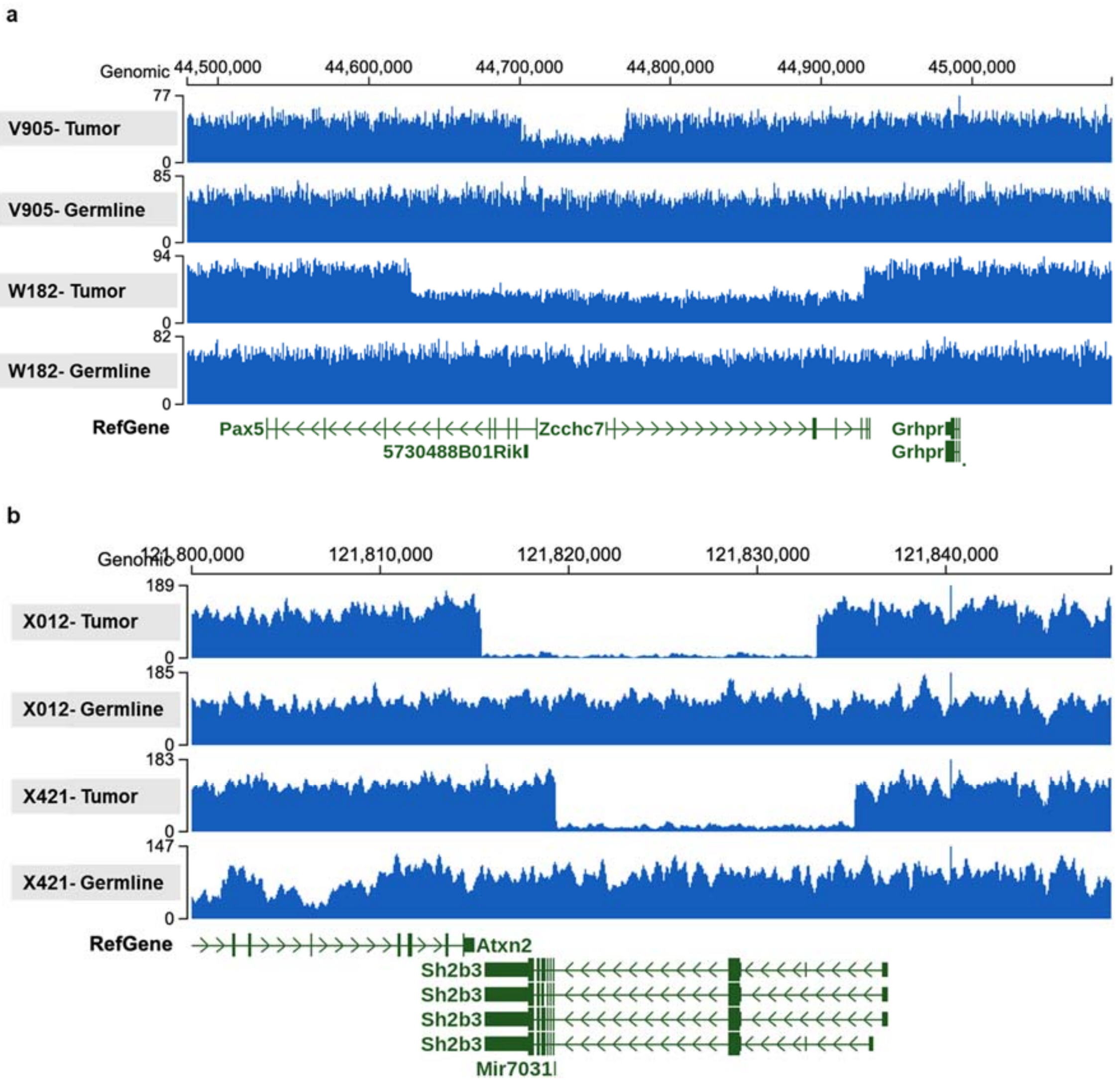

a
Genomic
44,500,000
44,600,000
44,700,000
44,800,000
44,900,000
45,000,000
77
V905- Tumor
0
85
V905- Germline
0
94
W182- Tumor
0
82
W182- Germline
0
RefGene
Pax5
Zcchc7
Grhpr
Grhpr
5730488B01Rik
b
Genomic
121,800,000
121,810,000
121,820,000
121,830,000
121,840,000
189
X012- Tumor
0
185
X012- Germline
0
183
X421- Tumor
0
147
X421- Germline
0
RefGene
Atxn2
Sh2b3
Sh2b3
Sh2b3
Sh2b3
Mir7031

c

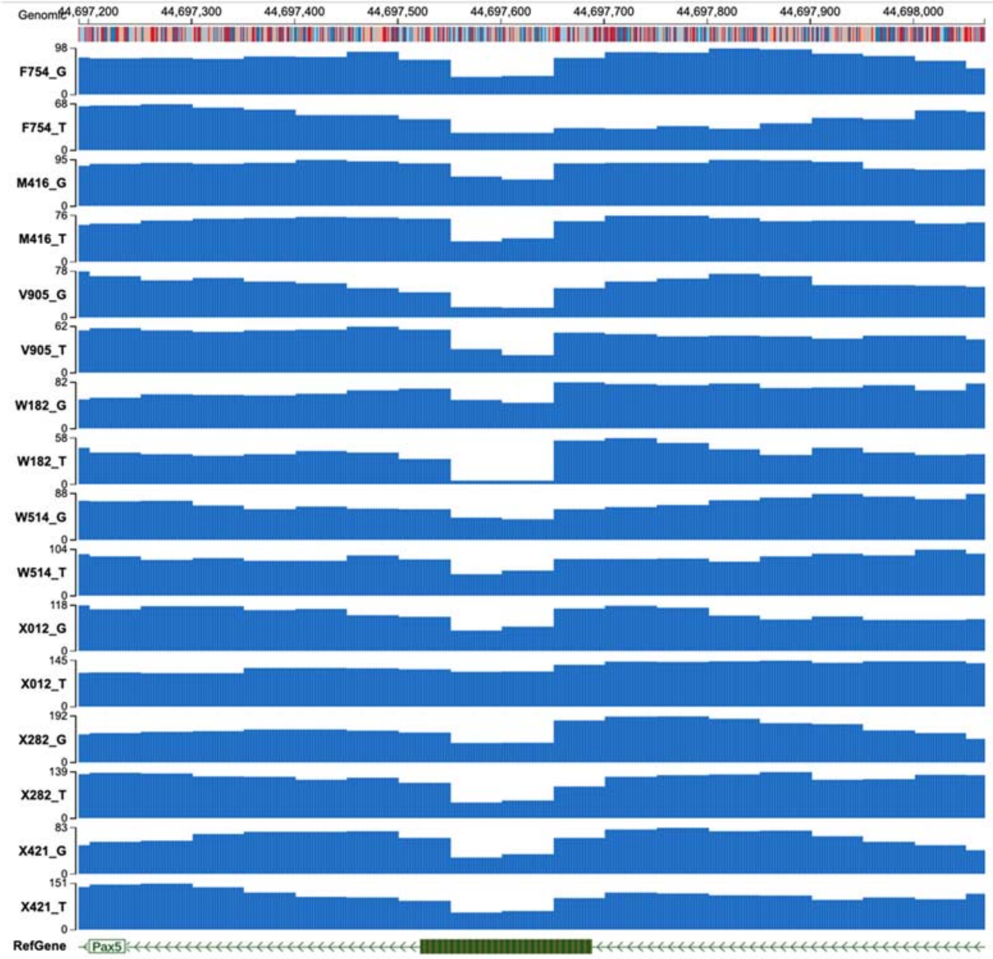

Supplementary Fig. S17: Visualization of somatic mutation in leukemic *Pax5*$^{+/-}$;*Myd88*$^{+/-}$ mice. Genome browser visualization of tumor DNA (T) and germline DNA (G) from tumor-bearing *Pax5*$^{+/-}$;*Myd88*$^{+/-}$ mice **a)** Somatic *Pax5* gene deletions identified by WGS in leukemic *Pax5*$^{+/-}$;*Myd88*$^{+/-}$ mice (V905 and W182). **b)** Somatic *Sh2b3* gene deletions identified by WGS in leukemic *Pax5*$^{+/-}$;*Myd88*$^{+/-}$ mice (X012 and X421). **c)** Validation of the germline *Pax5* exon 2 deletion by WGS from all the *Pax5*$^{+/-}$;*Myd88*$^{+/-}$ mice analysed by WGS. In the *Pax5*$^{+/-}$ mice the exon 2 of the *Pax5* gene (which is indispensable for DNA binding) was replaced by the Escherichia coli lac Z and neomycin resistance genes. WGS: whole-genome sequencing.

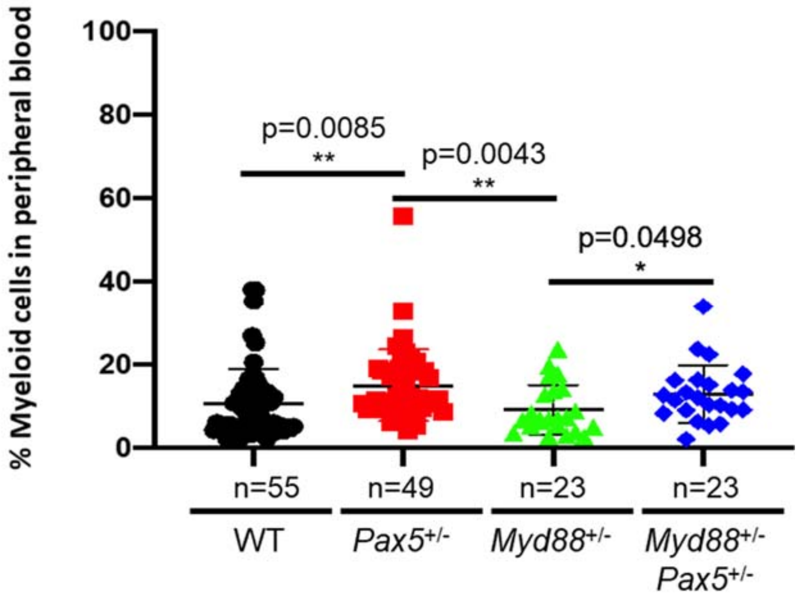

Supplementary Fig. S18: *Myd88$^{+/-}$;Pax5$^{+/-}$* mice present similar myeloid cells levels than *Pax5$^{+/-}$* mice. Percentage of myeloid cells in the peripheral blood of Myd88+/-; Pax5+/- (n=23) and Myd88+/- (n=23) mice compared with age-matched Pax5+/- (n=49) and WT (n=55) mice. Error bars represent the mean and SD of each group. For significant differences, p-values corresponding to unpaired t-test (two-tailed) are shown. Source data are provided as a Source Data file.

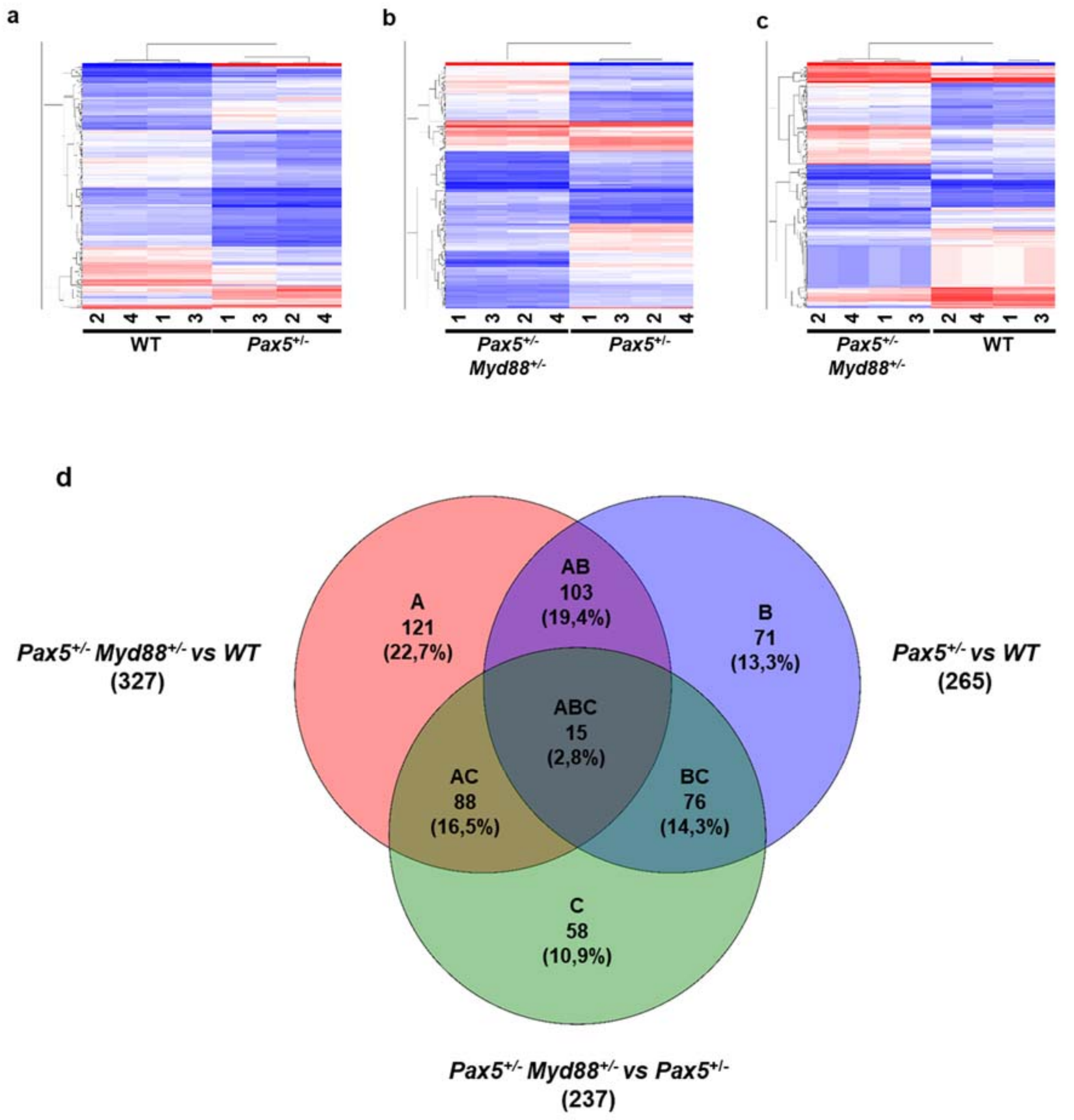

Supplementary Fig. S19: Gene expression analysis of healthy proB cells from WT, *Pax5*$^{+/-}$, and *Pax5*$^{+/-}$;*Myd88*$^{+/-}$mice. a) Unsupervised heatmap showing the differentially expressed genes (n=265) between healthy proB cells from *Pax5*$^{+/-}$ mice (n=4) and WT mice (n=4). **b)** Unsupervised heatmap showing the differentially expressed genes (n=237) between healthy proB cells from *Pax5*$^{+/-}$;*Myd88*$^{+/-}$ mice (n=4) and *Pax5*$^{+/-}$ mice (n=4). **c)** Unsupervised heatmap showing the differentially expressed genes (n=327) between healthy proB cells from *Pax5*$^{+/-}$;*Myd88*$^{+/-}$ mice (n=4) and *WT* mice (n=4). The significance analysis of microarrays was defined by a p-value<0.05 and fold change: FC<-2 or FC>2. **d**) Venn diagram showing the overlapped genes of the differentially expressed genes between the three groups analyzed.

a

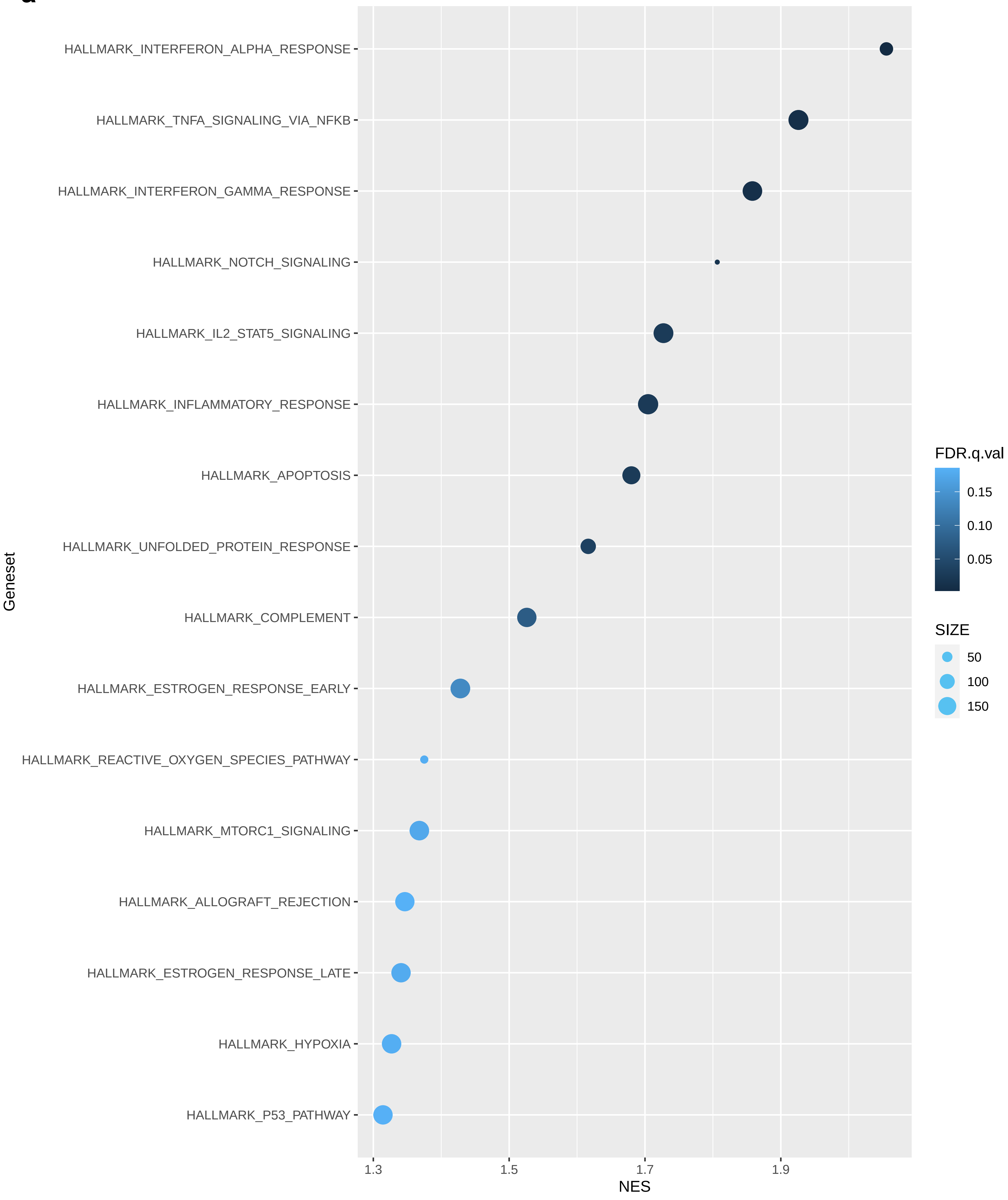

HALLMARK_INTERFERON_ALPHA_RESPONSE
HALLMARK_TNFA_SIGNALING_VIA_NFKB
HALLMARK_INTERFERON_GAMMA_RESPONSE
HALLMARK_NOTCH_SIGNALING
HALLMARK_IL2_STAT5_SIGNALING
HALLMARK_INFLAMMATORY_RESPONSE
HALLMARK_APOPTOSIS
HALLMARK_UNFOLDED_PROTEIN_RESPONSE
HALLMARK_COMPLEMENT
HALLMARK_ESTROGEN_RESPONSE_EARLY
HALLMARK_REACTIVE_OXYGEN_SPECIES_PATHWAY
HALLMARK_MTORC1_SIGNALING
HALLMARK_ALLOGRAFT_REJECTION
HALLMARK_ESTROGEN_RESPONSE_LATE
HALLMARK_HYPOXIA
HALLMARK_P53_PATHWAY
Geneset
1.3
1.5
1.7
1.9
NES
FDR.q.val
0.15
0.10
0.05
SIZE
50
100
150

b

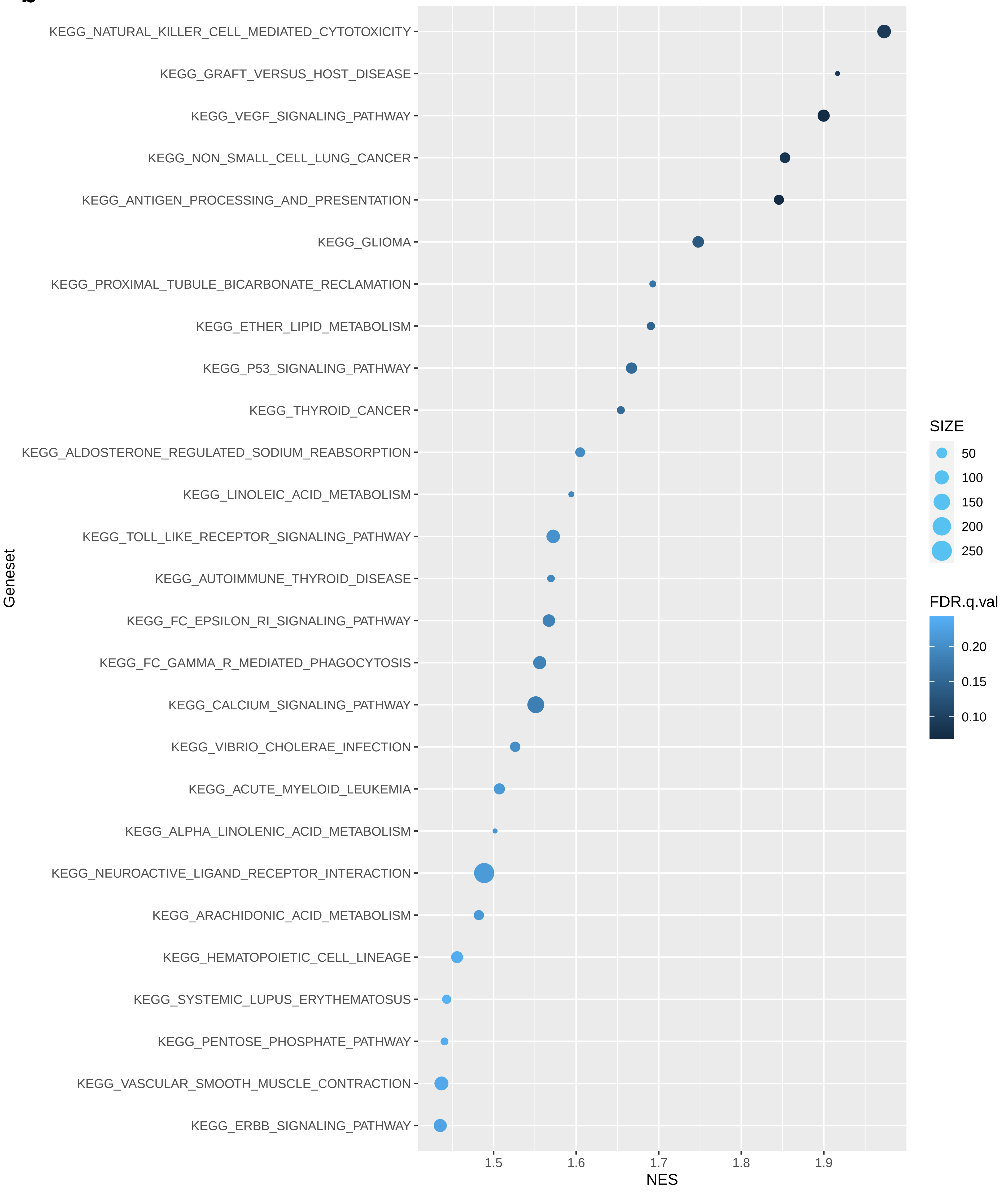
KEGG_NATURAL_KILLER_CELL_MEDIATED_CYTOTOXICITY
KEGG_GRAFT_VERSUS_HOST_DISEASE
KEGG_VEGF_SIGNALING_PATHWAY
KEGG_NON_SMALL_CELL_LUNG_CANCER
KEGG_ANTIGEN_PROCESSING_AND_PRESENTATION
KEGG_GLIOMA
KEGG_PROXIMAL_TUBULE_BICARBONATE_RECLAMATION
KEGG_ETHER_LIPID_METABOLISM
KEGG_P53_SIGNALING_PATHWAY
KEGG_THYROID_CANCER
KEGG_ALDOSTERONE_REGULATED_SODIUM_REABSORPTION
KEGG_LINOLEIC_ACID_METABOLISM
KEGG_TOLL_LIKE_RECEPTOR_SIGNALING_PATHWAY
KEGG_AUTOIMMUNE_THYROID_DISEASE
KEGG_FC_EPSILON_RI_SIGNALING_PATHWAY
KEGG_FC_GAMMA_R_MEDIATED_PHAGOCYTOSIS
KEGG_CALCIUM_SIGNALING_PATHWAY
KEGG_VIBRIO_CHOLERAE_INFECTION
KEGG_ACUTE_MYELOID_LEUKEMIA
KEGG_ALPHA_LINOLENIC_ACID_METABOLISM
KEGG_NEUROACTIVE_LIGAND_RECEPTOR_INTERACTION
KEGG_ARACHIDONIC_ACID_METABOLISM
KEGG_HEMATOPOIETIC_CELL_LINEAGE
KEGG_SYSTEMIC_LUPUS_ERYTHEMATOSUS
KEGG_PENTOSE_PHOSPHATE_PATHWAY
KEGG_VASCULAR_SMOOTH_MUSCLE_CONTRACTION
KEGG_ERBB_SIGNALING_PATHWAY
Geneset
1.5
1.6
1.7
1.8
1.9
NES
SIZE
50
100
150
200
250
FDR.q.val
0.20
0.15
0.10

c

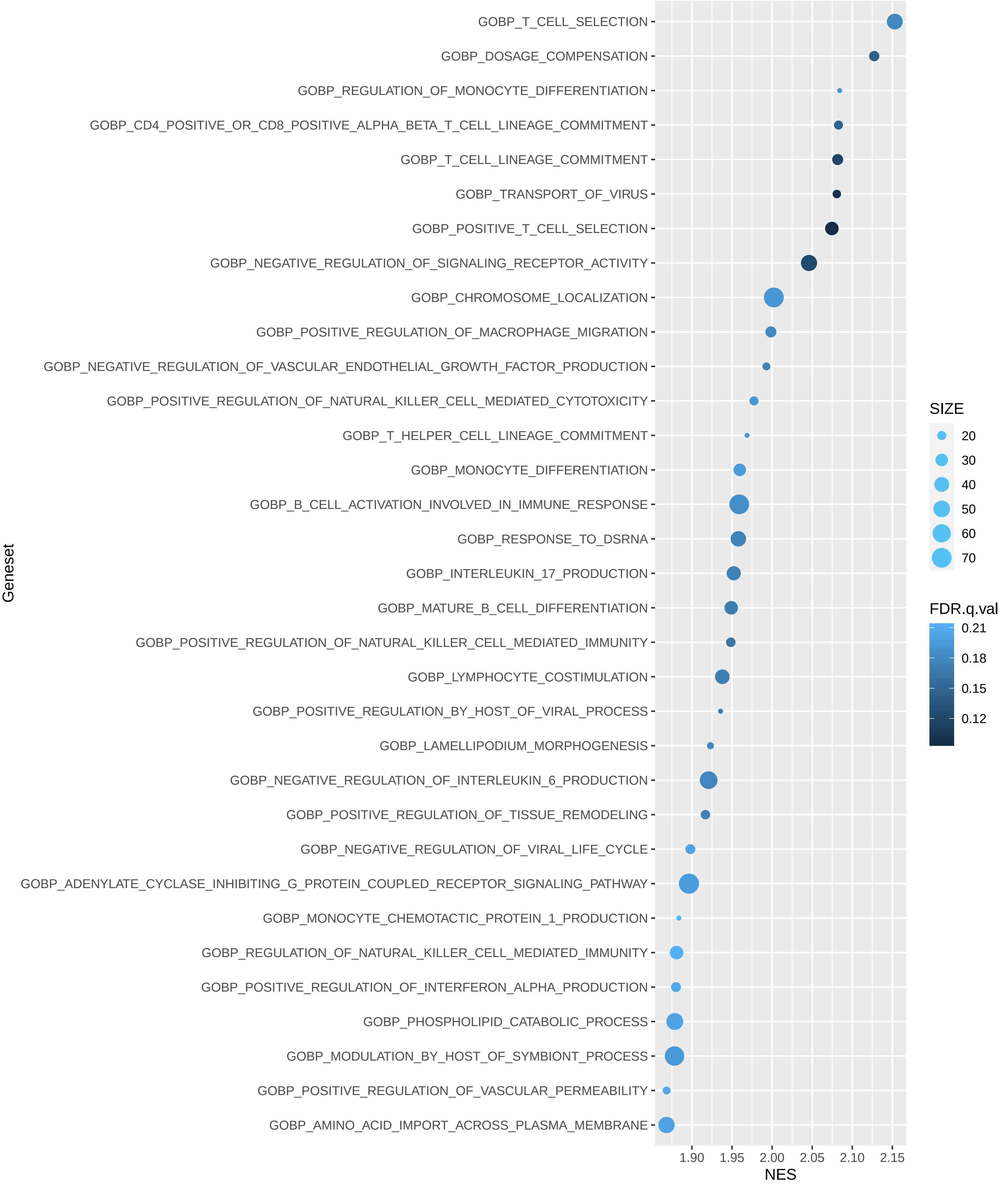

GOBP_T_CELL_SELECTION
GOBP_DOSAGE_COMPENSATION
GOBP_REGULATION_OF_MONOCYTE_DIFFERENTIATION
GOBP_CD4_POSITIVE_OR_CD8_POSITIVE_ALPHA_BETA_T_CELL_LINEAGE_COMMITMENT
GOBP_T_CELL_LINEAGE_COMMITMENT
GOBP_TRANSPORT_OF_VIRUS
GOBP_POSITIVE_T_CELL_SELECTION
GOBP_NEGATIVE_REGULATION_OF_SIGNALING_RECEPTOR_ACTIVITY
GOBP_CHROMOSOME_LOCALIZATION
GOBP_POSITIVE_REGULATION_OF_MACROPHAGE_MIGRATION
GOBP_NEGATIVE_REGULATION_OF_VASCULAR_ENDOTHELIAL_GROWTH_FACTOR_PRODUCTION
GOBP_POSITIVE_REGULATION_OF_NATURAL_KILLER_CELL_MEDIATED_CYTOTOXICITY
GOBP_T_HELPER_CELL_LINEAGE_COMMITMENT
GOBP_MONOCYTE_DIFFERENTIATION
GOBP_B_CELL_ACTIVATION_INVOLVED_IN_IMMUNE_RESPONSE
GOBP_RESPONSE_TO_DSRNA
GOBP_INTERLEUKIN_17_PRODUCTION
GOBP_MATURE_B_CELL_DIFFERENTIATION
GOBP_POSITIVE_REGULATION_OF_NATURAL_KILLER_CELL_MEDIATED_IMMUNITY
GOBP_LYMPHOCYTE_COSTIMULATION
GOBP_POSITIVE_REGULATION_BY_HOST_OF_VIRAL_PROCESS
GOBP_LAMELLIPODIUM_MORPHOGENESIS
GOBP_NEGATIVE_REGULATION_OF_INTERLEUKIN_6_PRODUCTION
GOBP_POSITIVE_REGULATION_OF_TISSUE_REMODELING
GOBP_NEGATIVE_REGULATION_OF_VIRAL_LIFE_CYCLE
GOBP_ADENYLATE_CYCLASE_INHIBITING_G_PROTEIN_COUPLED_RECEPTOR_SIGNALING_PATHWAY
GOBP_MONOCYTE_CHEMOTACTIC_PROTEIN_1_PRODUCTION
GOBP_REGULATION_OF_NATURAL_KILLER_CELL_MEDIATED_IMMUNITY
GOBP_POSITIVE_REGULATION_OF_INTERFERON_ALPHA_PRODUCTION
GOBP_PHOSPHOLIPID_CATABOLIC_PROCESS
GOBP_MODULATION_BY_HOST_OF_SYMBIONT_PROCESS
GOBP_POSITIVE_REGULATION_OF_VASCULAR_PERMEABILITY
GOBP_AMINO_ACID_IMPORT_ACROSS_PLASMA_MEMBRANE
Geneset
1.90
1.95
2.00
2.05
2.10
2.15
NES
SIZE
20
30
40
50
60
70
FDR.q.val
0.21
0.18
0.15
0.12

Supplementary Fig. S20: Gene expression enrichment analysis of healthy proB cells from Pax5$^{+/-}$;Myd88$^{+/-}$ and compared with Pax5$^{+/-}$ proB cells. Gene Set Enrichment Analyses were performed using the gene sets from hallmark collection database **a)**, canonical pathways gene sets derived from the KEGG pathway database **b)** and gene sets derived from the GO Biological Process Ontology **c)** from MSigDB[4-6]. This analysis identifies significant enrichment of several pathways in Myd88$^{+/-}$Pax5$^{+/-}$ healthy pro B cells plotted on the y-axis. On the x-axis, the NES (Normalized Enrichment Score) value is represented for each gene set. The corresponding FDR (False Discovery Rate) value is represented in a blue color scale and the dot size depicts the gene count. Source data are provided as a Source Data file.

a

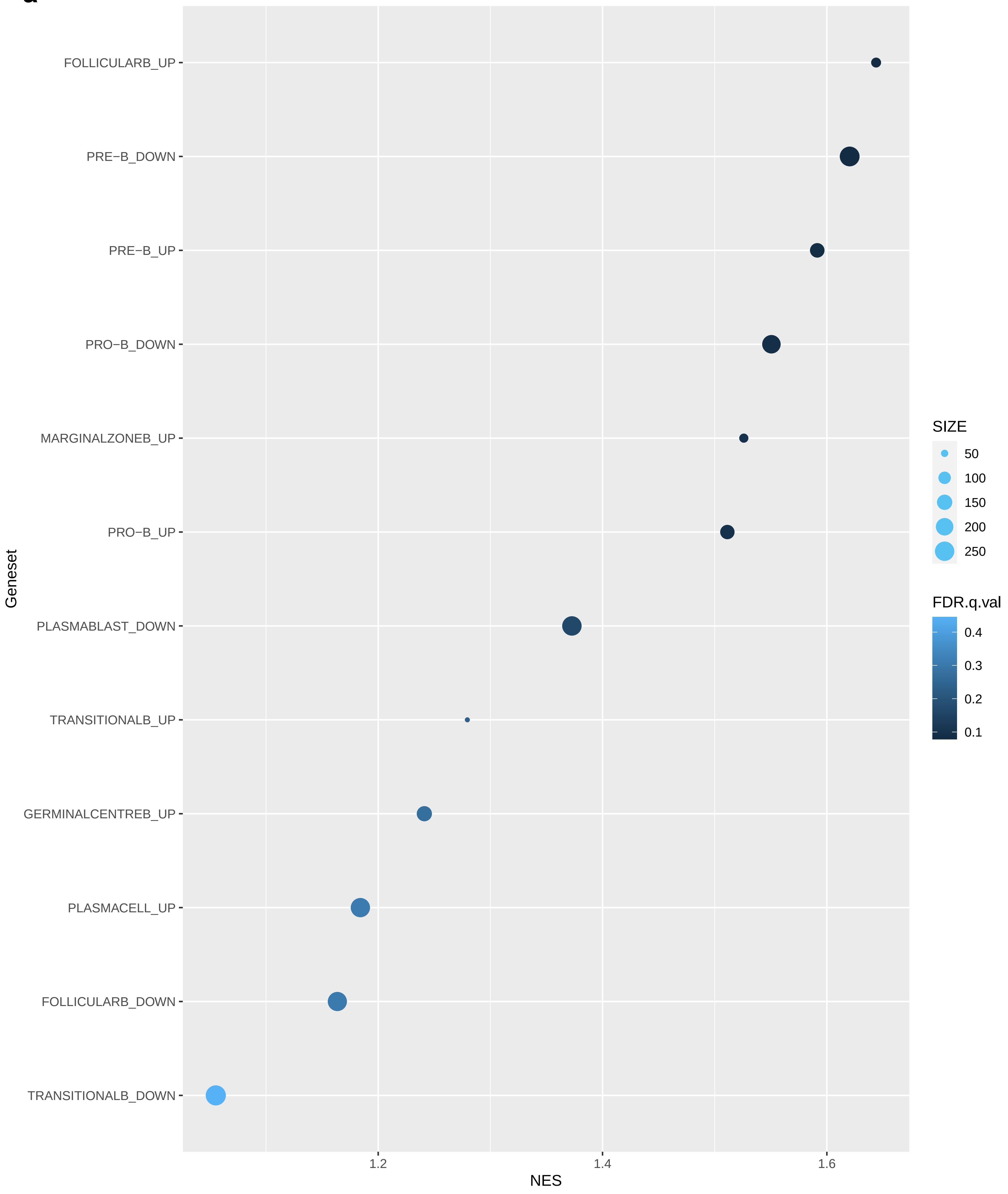

FOLLICULARB_UP
PRE−B_DOWN
PRE−B_UP
PRO−B_DOWN
MARGINALZONEB_UP
PRO−B_UP
PLASMABLAST_DOWN
TRANSITIONALB_UP
GERMINALCENTREB_UP
PLASMACELL_UP
FOLLICULARB_DOWN
TRANSITIONALB_DOWN
Geneset
1.2
1.4
1.6
NES
SIZE
50
100
150
200
250
FDR.q.val
0.4
0.3
0.2
0.1

b

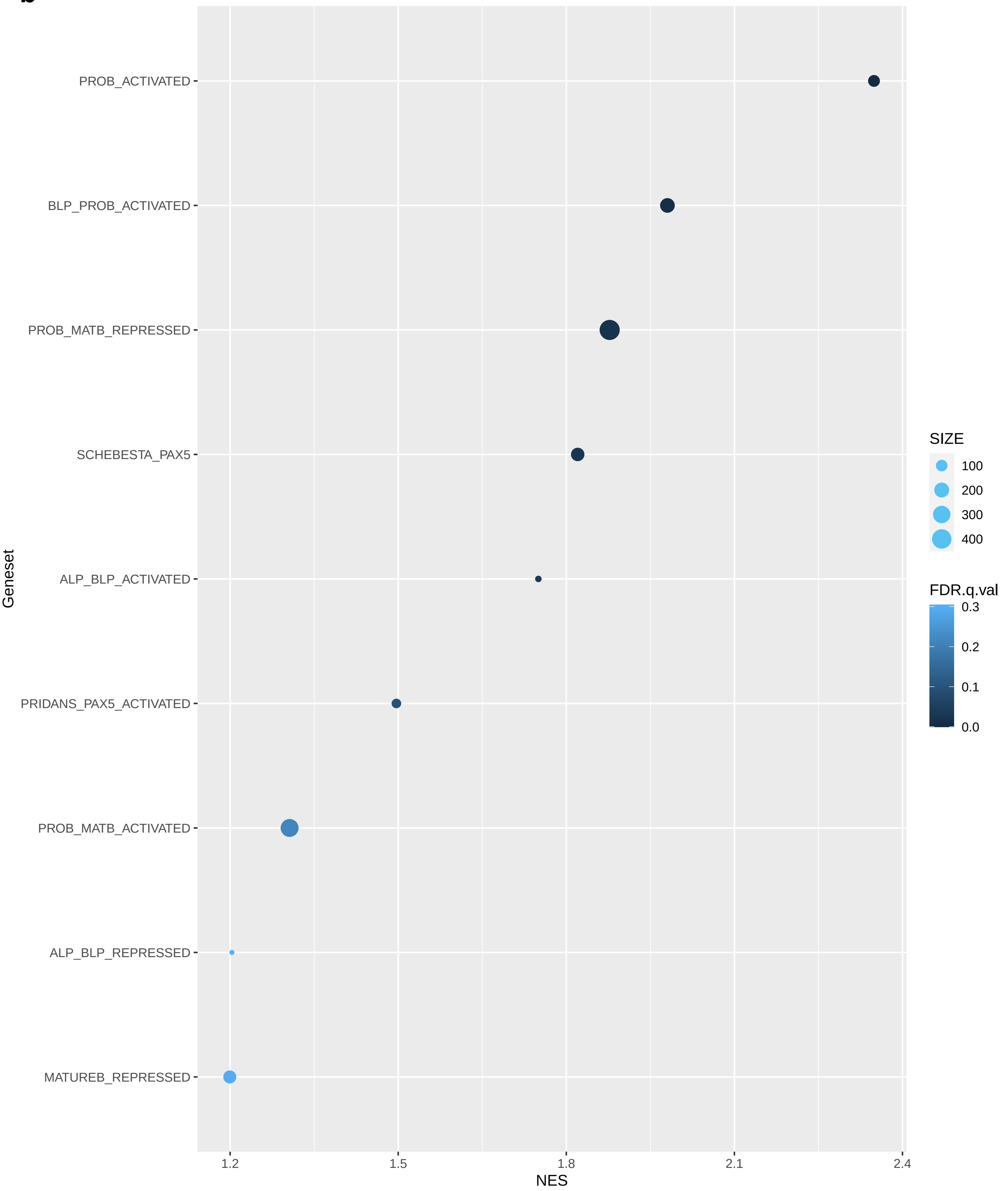

PROB_ACTIVATED
BLP_PROB_ACTIVATED
PROB_MATB_REPRESSED
SCHEBESTA_PAX5
ALP_BLP_ACTIVATED
PRIDANS_PAX5_ACTIVATED
PROB_MATB_ACTIVATED
ALP_BLP_REPRESSED
MATUREB_REPRESSED
Geneset
1.2
1.5
1.8
2.1
2.4
NES
SIZE
100
200
300
400
FDR.q.val
0.3
0.2
0.1
0.0

Supplementary Fig. S21: Impact of *Myd88* downregulation in gene expression of *Pax5*$^{+/-}$ healthy proB cells. a) Gene set enrichment analyses showing the enriched murine B cell developmental stages, plotted on the y-axis, in *Pax5*$^{+/-}$;*Myd88*$^{+/-}$ proB cells from healthy mice. 16 different developmental stages were assessed and gene sets were extracted from Green et al.[7] **b)** Gene-sets of Pax5-regulated genes, plotted on the y-axis, were more enriched in *Pax5*$^{+/-}$;*Myd88*$^{+/-}$ proB cells that in *Pax5*$^{+/-}$ healthy proB cells. Genesets were extracted from Revilla et al.[8] and Schebesta et al.[9]. On the x-axis, the NES (Normalized Enrichment Score) value is represented for each gene set. The corresponding FDR (False Discovery Rate) value is represented in a blue color scale and the dot size depicts the gene count. (BLP: B-cell-biased lymphoid progenitors; ALP: all-lymphoid progenitors; MATB: mature B cells). Source data are provided as a Source Data file.

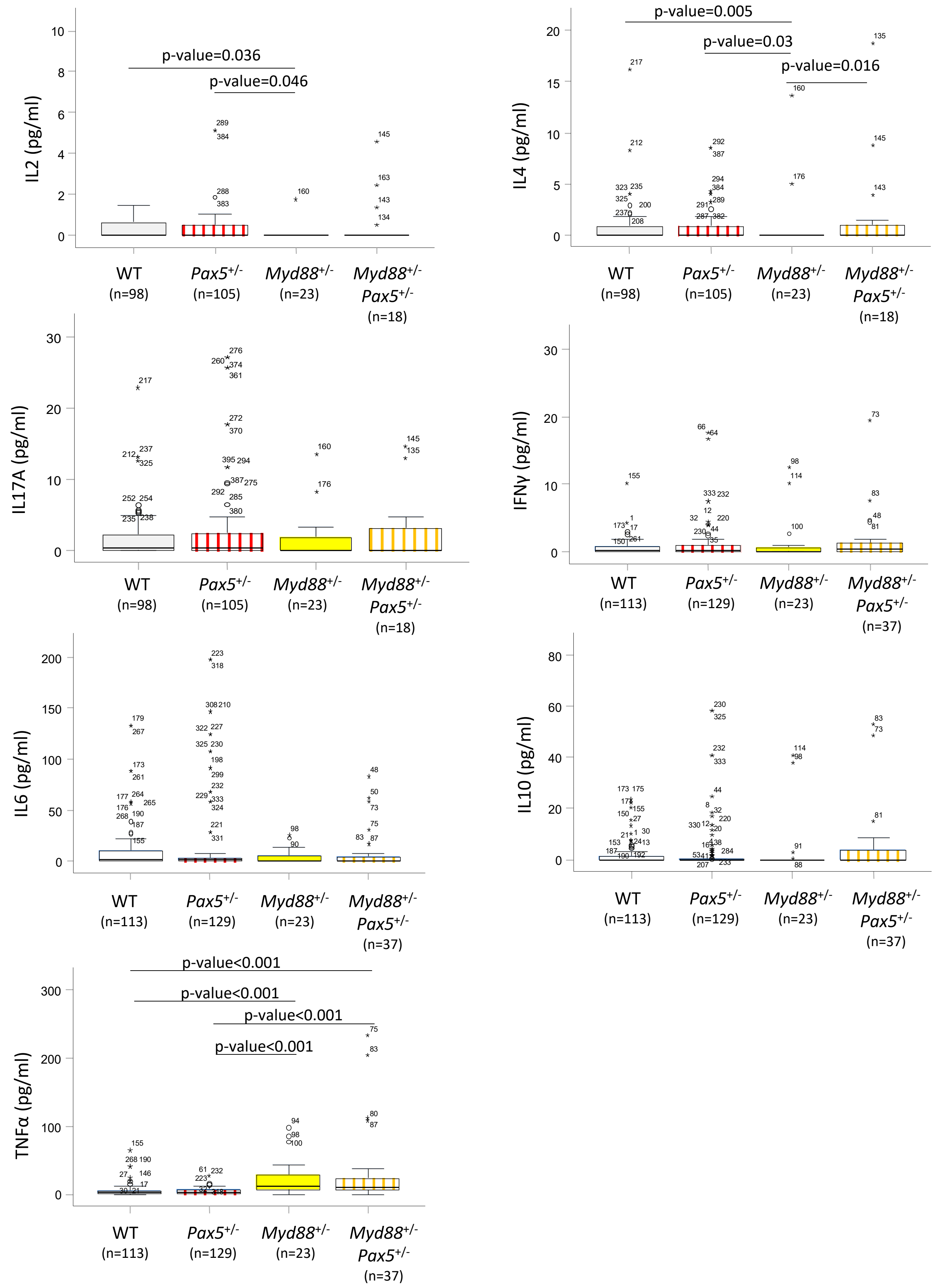

Supplementary Fig. S22: Inflammatory cytokines levels in *Pax5*$^{+/-}$, *Myd88*$^{+/-}$*;Pax5*$^{+/+}$ and *Myd88*$^{+/-}$*;Pax5*$^{+/-}$ mice. IL-2, IL-4, IL-6, IL-10, IL-17a, IFN $\gamma$and TNF αserum levels in WT, *Pax5*$^{+/-}$, *Myd88*$^{+/-}$*;Pax5*$^{+/+}$ and *Myd88*$^{+/-}$*;Pax5*$^{+/-}$ mice. The different cytokines' serum levels were at different ages of the mice. Error bars represent the mean and SD. For significant differences between groups, Mann-Whitney p-values are indicated in each case. When comparing each cytokine using Kruskal-Wallis test, significant p-values are only significant for IL4 (p=0.044) and TNF α(p<0.001). Source data are provided as a Source Data file.

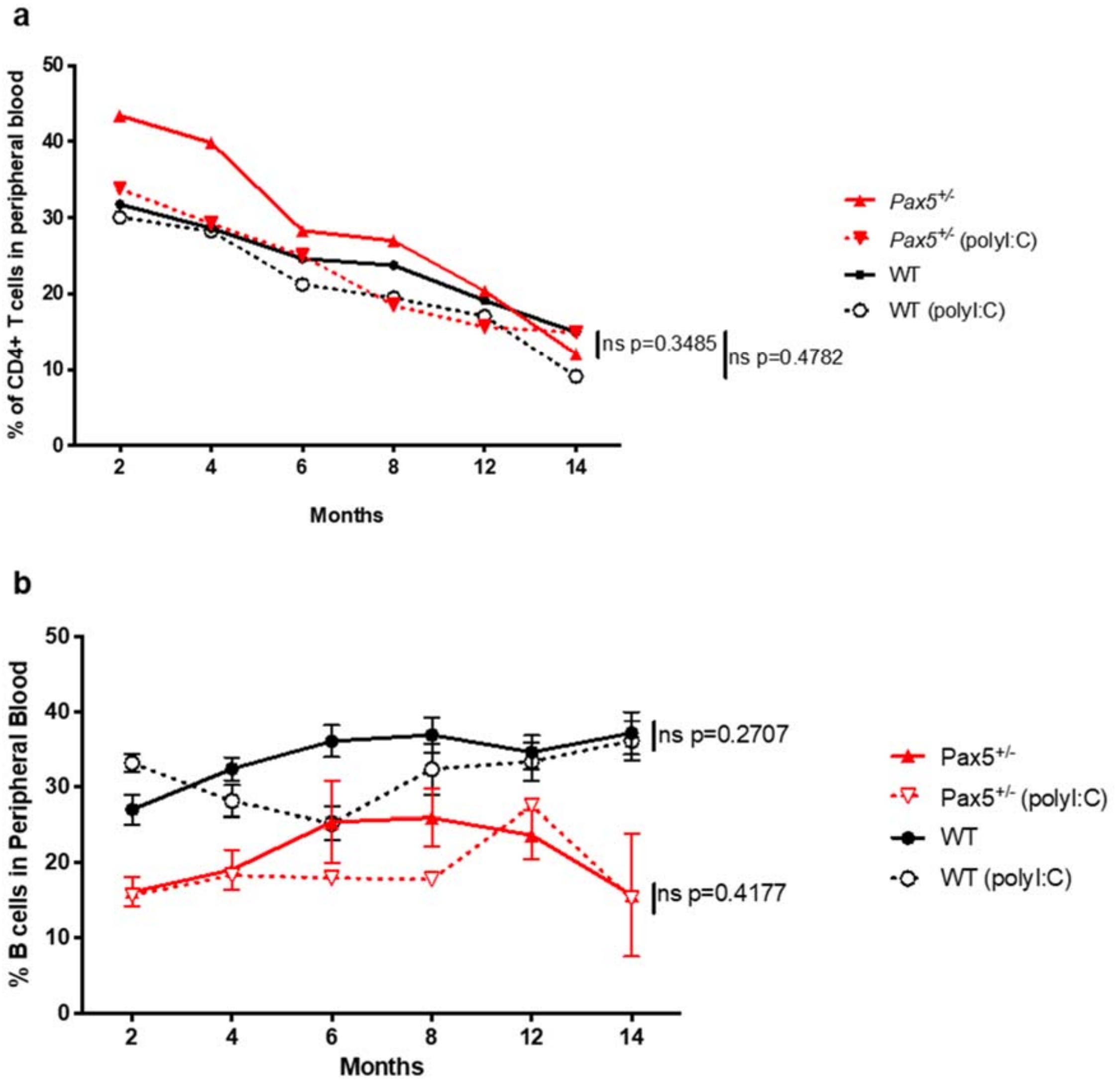

Supplementary Fig. S23: Percentages of CD4+ T (a) and B (b) cells in peripheral blood of *Pax5*$^{+/-}$ and WT mice treated with poly(I:C) compared to untreated mice. Each point represents the mean of the levels of the different populations in all mice in each group for the different time points (n=31 for *Pax5*$^{+/-}$ poly(I:C)-treated mice, n=8 for *Pax5*$^{+/-}$ untreated mice, n=14 for WT poly(I:C) treated mice and n=24 for WT untreated mice). Error bars represent the mean and SD of each group. P-values corresponding to unpaired t-test (two-tailed) are shown. Source data are provided as a Source Data file.

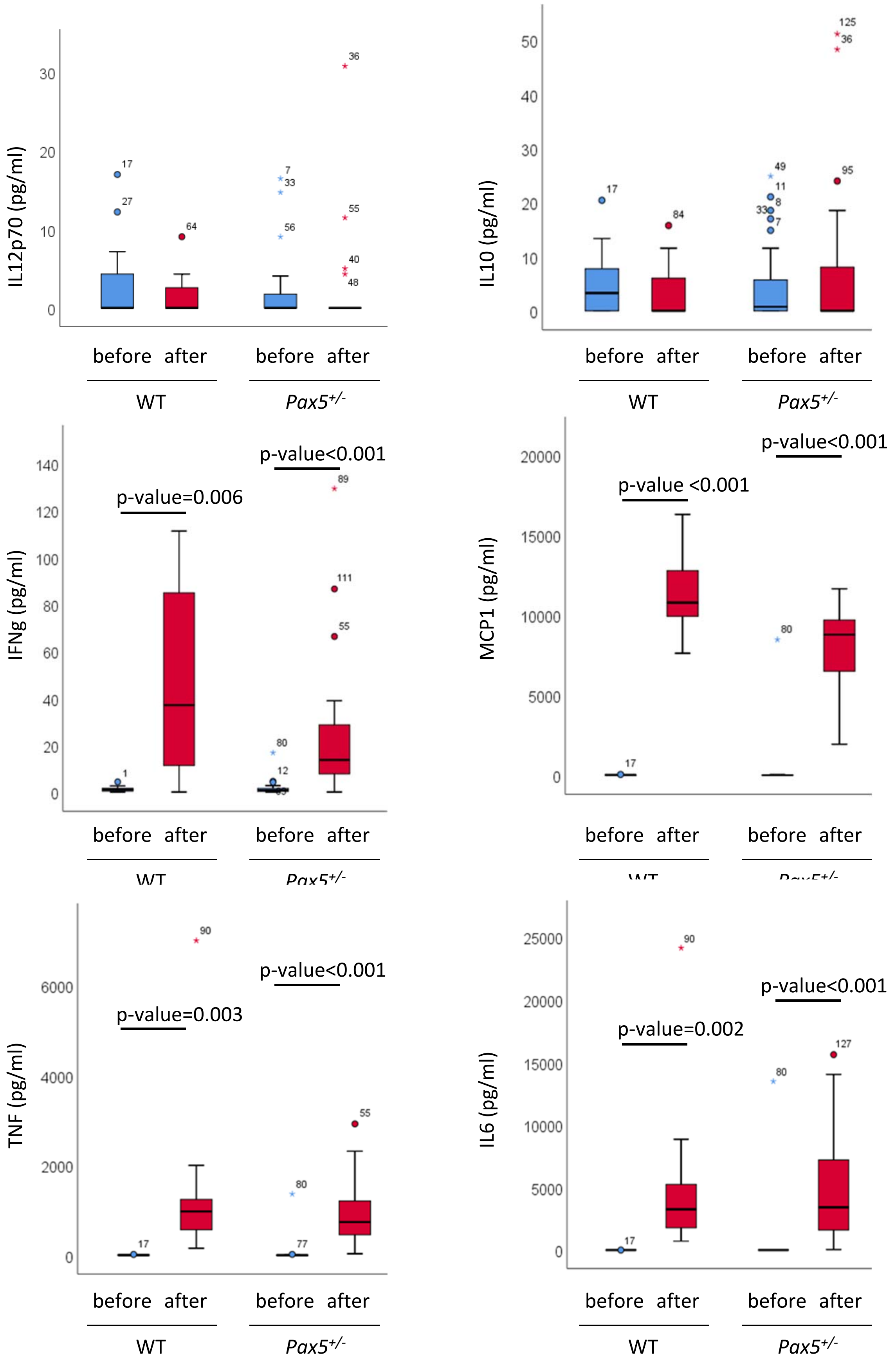

Supplementary Fig. S24: Impact of poly(I:C) treatment on the serum cytokines. The plots showed how poly(I:C) treatment changed the levels of some inflammatory cytokines in the serum of Pax5+/- (n=30) and WT (n=14) mice. TNF-alpha, INF-gamma, MCP1, and IL6 levels showed an increase in the serum of the mice after poly(I:C) treatment. IL12p70 and IL10 did not show changes in their levels. The different cytokines' serum levels were measured before and 3 hours after the administration of the poly(I:C). Error bars represent the mean and SD. For the significant differences, paired t-test p-values are indicated in each case. Source data are provided as a Source Data file.

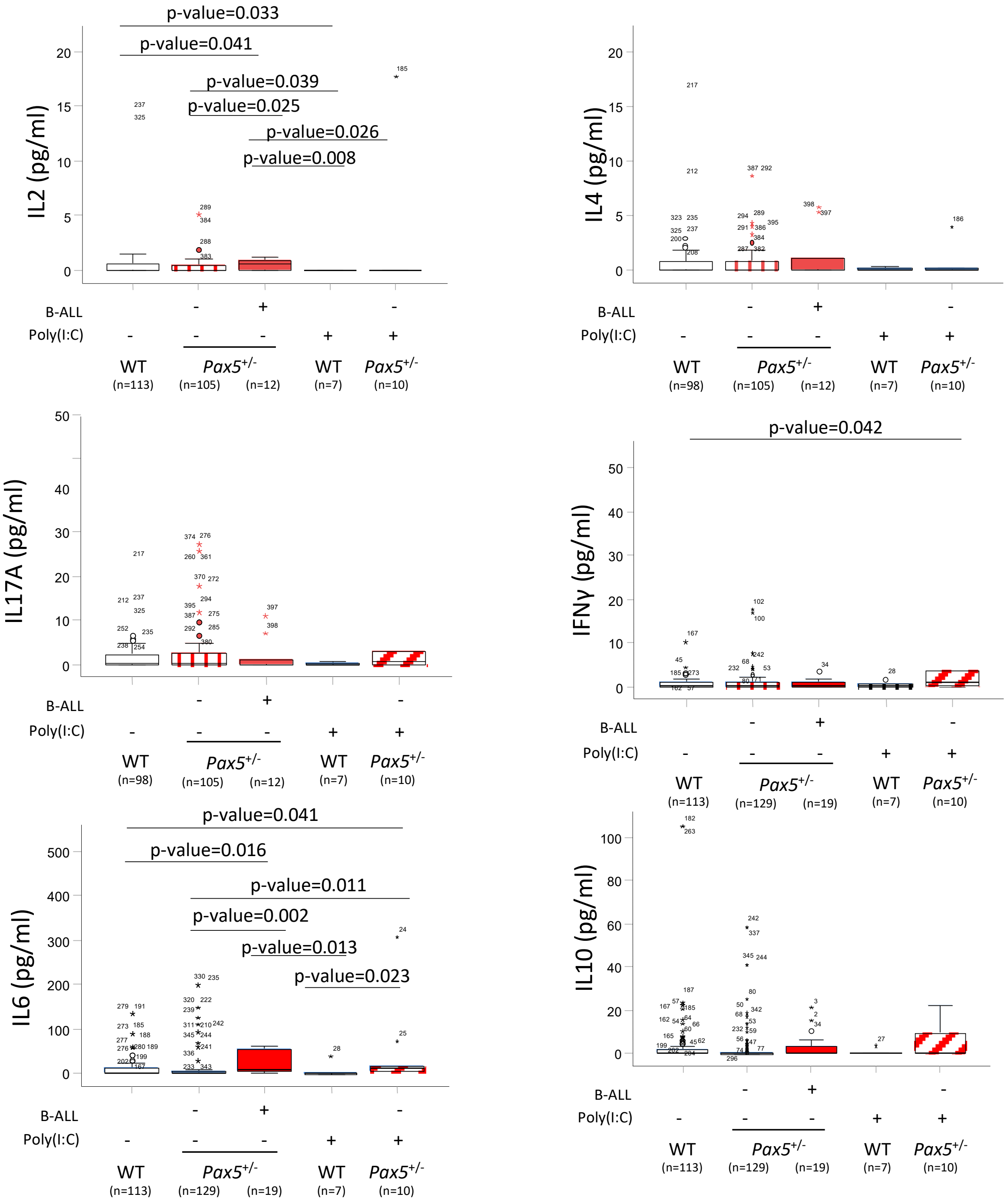

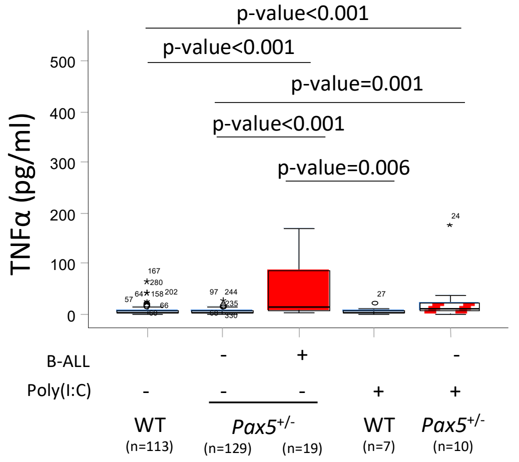

Supplementary Fig. S25: Inflammatory cytokines levels in *Pax5*$^{+/-}$ and WT mice treated with poly(I:C) compared with untreated *Pax5*$^{+/-}$ and WT mice. IL-2, IL-4, IL-6, IL-10, IL-17a, IFN γand TNF αserum levels in WT and *Pax5*$^{+/-}$ mice treated with poly(I:C) compared with WT and *Pax5*$^{+/-}$ mice non-treated. The different cytokines' serum levels were at different ages of the mice. Error bars represent the mean and SD. For significant differences between groups, Mann-Whitney p-values are indicated in each case. When comparing each cytokine using Kruskal-Wallis test, significant p-values are only significant for IL2 (p=0.013), IL6 (p=0.001) and TNF α(p<0.001). Source data are provided as a Source Data file.

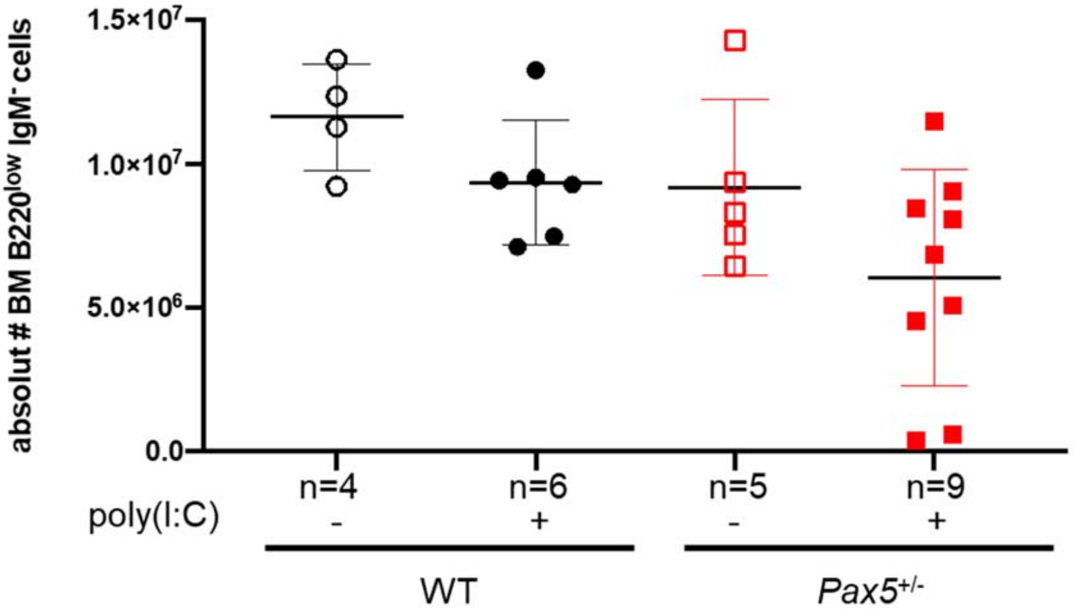

Supplementary Figure S26. Poly(I:C) treatment does not reduce the preleukemic compartment in the bone marrow of *Pax5*$^{+/-}$ mice. Absolute numbers of preB and proB cells (B220^{low} IgM^{-}) in the bone marrow (BM) of *Pax5*$^{+/-}$ (n=9) and WT (n=5) poly(I:C)-treated mice compared with age-matched *Pax5*$^{+/-}$ (n=5) and WT (n=4) mice treated with PBS. Error bars represent the mean and SD of each group. For significant differences, p-values corresponding to unpaired t-test (two-tailed) are shown. Source data are provided as a Source Data file.

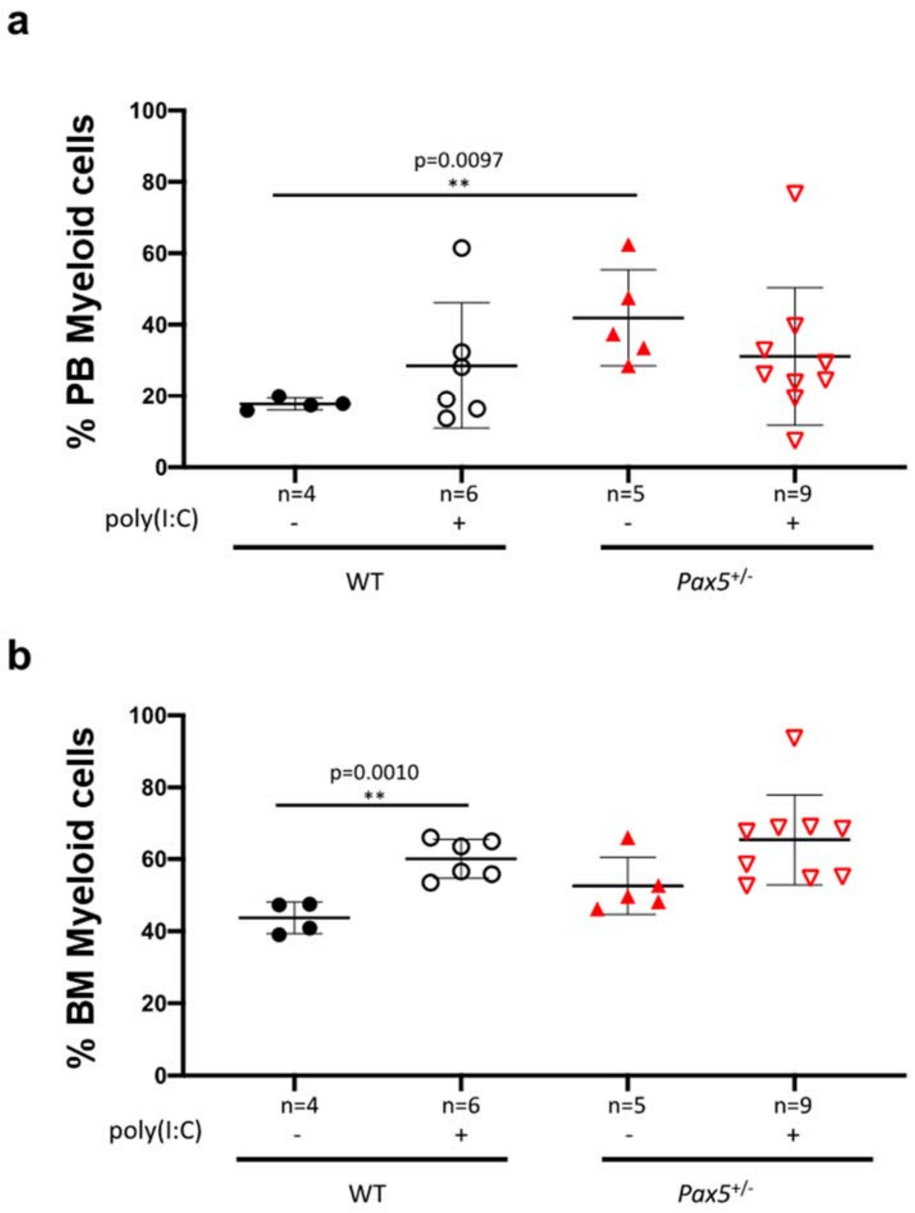

Supplementary Figure S27. *Pax5*$^{+/-}$ mice treated with poly(I:C) present similar myeloid cells levels than untreated *Pax5*$^{+/-}$ mice. a) Percentage of myeloid cells (Gr1^{+} Mac1^{+}) in the peripheral blood (PB) of *Pax5*$^{+/-}$ (n=9) and WT (n=5) poly(I:C)-treated mice compared with age-matched *Pax5*$^{+/-}$ (n=5) and WT (n=4) mice treated with PBS. Error bars represent the mean and SD of each group. For significant differences, p-values corresponding to unpaired t-test (two-tailed) are shown. **b)** Percentage of myeloid cells (Gr1^{+} Mac1^{+}) in the bone marrow (BM) of *Pax5*$^{+/-}$ (n=9) and WT (n=5) poly(I:C)-treated mice compared with age-matched *Pax5*$^{+/-}$ (n=5) and WT (n=4) mice treated with PBS. Error bars represent the mean and SD of each group. For significant differences, p-values corresponding to unpaired t-test (two-tailed) are shown. Source data are provided as a Source Data file.

a

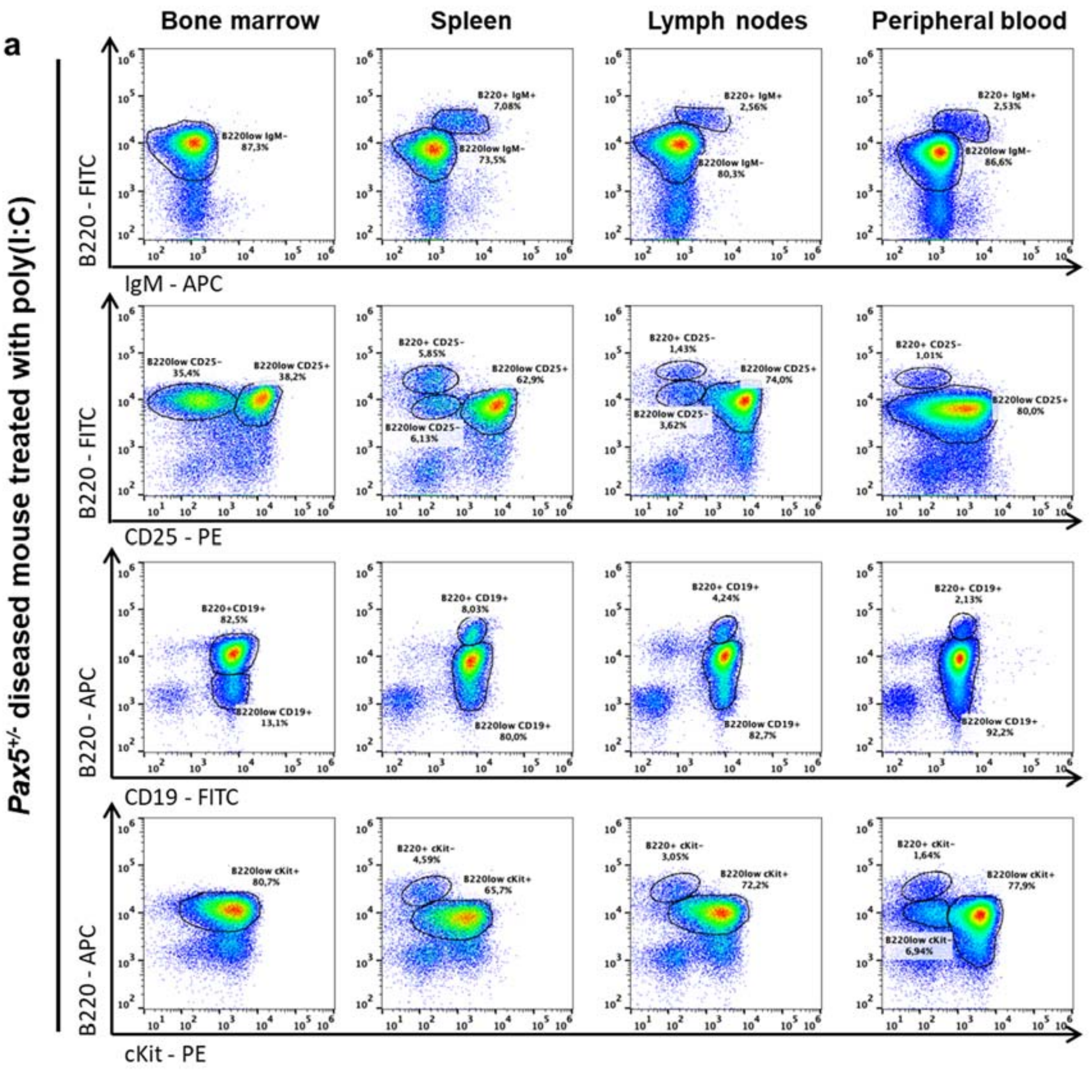

Bone marrow
Spleen
Lymph nodes
Peripheral blood
Pax5+/- diseased mouse treated with poly(I:C)
B220 - FITC
IgM - APC
CD25 - PE
B220 - APC
CD19 - FITC
cKit - PE

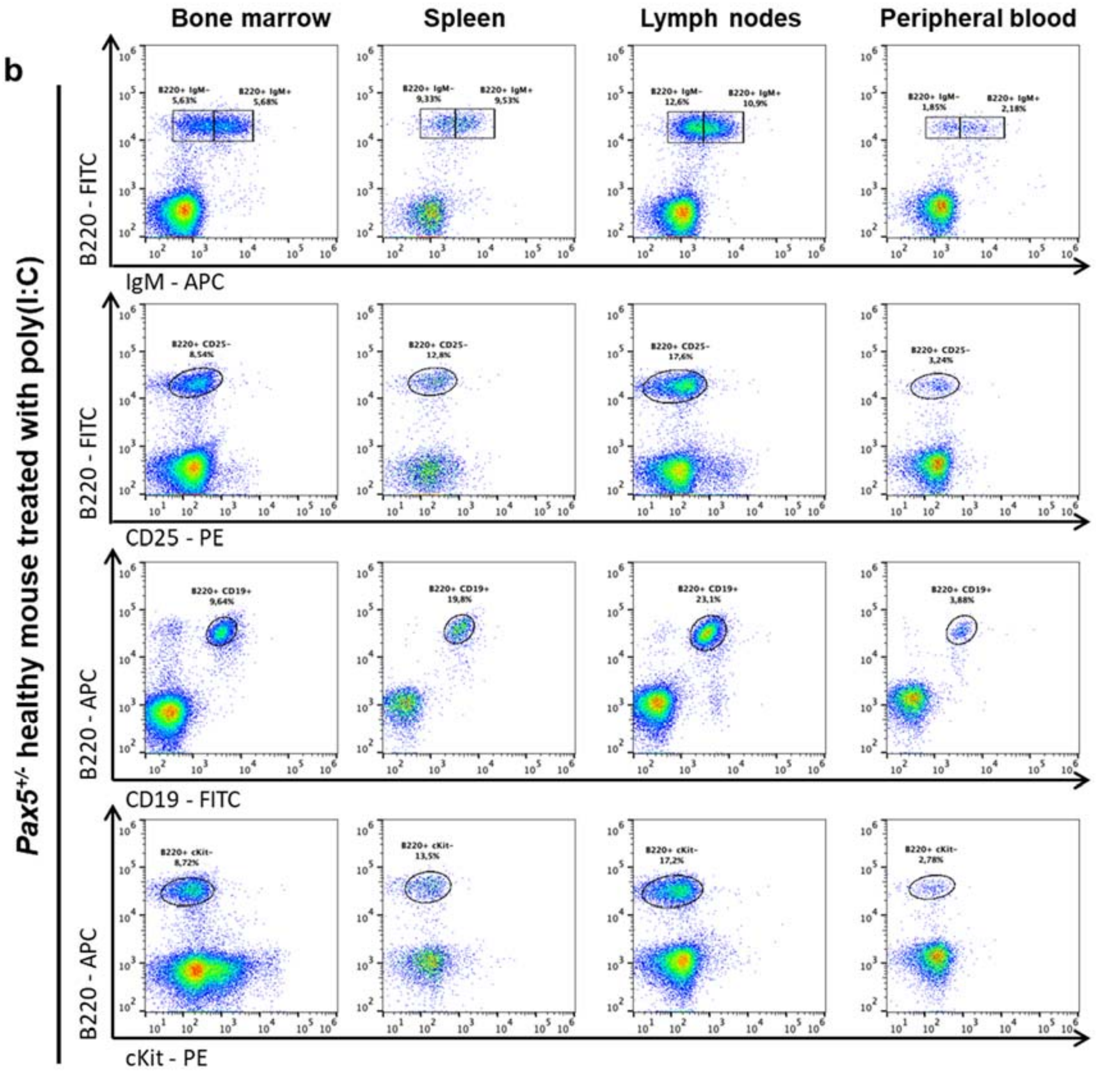
b
Bone marrow
Spleen
Lymph nodes
Peripheral blood
Pax5+/- healthy mouse treated with poly(I:C)
B220 - FITC
IgM - APC
CD25 - PE
B220 - APC
CD19 - FITC
cKit - PE
B220+ IgM-
5,63%
B220+ IgM+
5,68%
9,33%
9,53%
12,6%
10,9%
1,85%
2,18%
B220+ CD25-
8,54%
12,8%
17,6%
3,24%
B220+ CD19+
9,64%
19,8%
23,1%
3,88%
B220+ cKit-
8,72%
13,5%
17,2%
2,78%

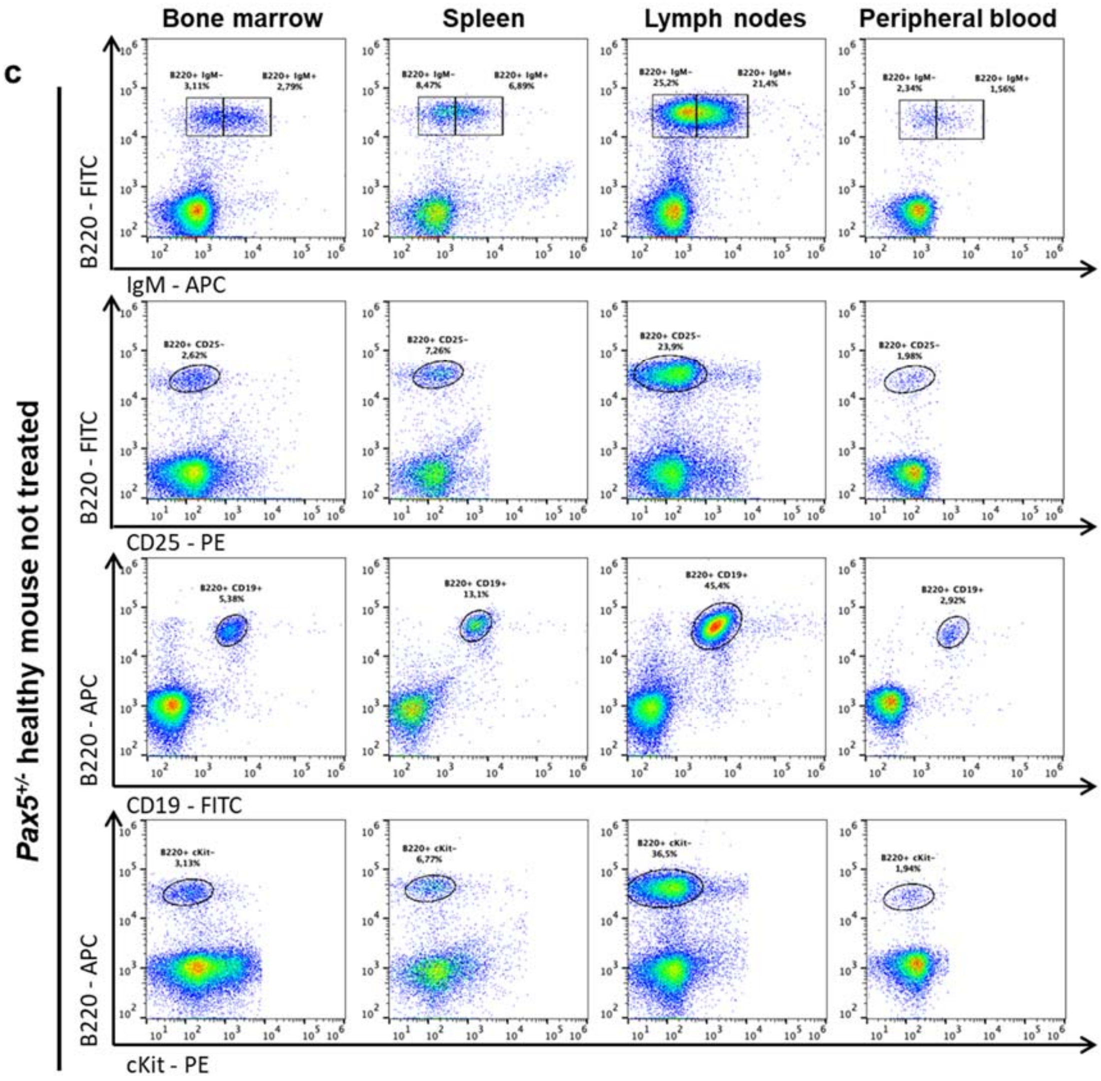

Supplementary Fig. S28: Phenotype characterization of leukemic cells from diseased *Pax5*$^{+/-}$ mice treated with poly(I:C). Flow cytometric analysis of hematopoietic subsets in diseased *Pax5*$^{+/-}$ mice treated with poly(I:C) (**a**), in a healthy *Pax5*$^{+/-}$ mice treated with poly(I:C) (**b**) and in a healthy untreated *Pax5*$^{+/-}$ mice (**c**), all of them exposed to common infections. Representative plots of cell subsets from bone marrow, spleen, lymph nodes and peripheral blood show the accumulation of malignant B cells in leukemic *Pax5*$^{+/-}$ mice compared to an aged healthy *Pax5*$^{+/-}$ mouse treated both with poly(I:C) and to a healthy untreated *Pax5*$^{+/-}$ mouse. Flow cytometric images in panel A are representative of 8 mice analysed that developed leukemia after poly(I:C) treatment. The leukemic cells showed a B220^{low} IgM^{-} CD25$^{+/-}$ CD19$^{+/-}$ cKit$^{+/-}$ phenotype and were detected only in the diseased mice.

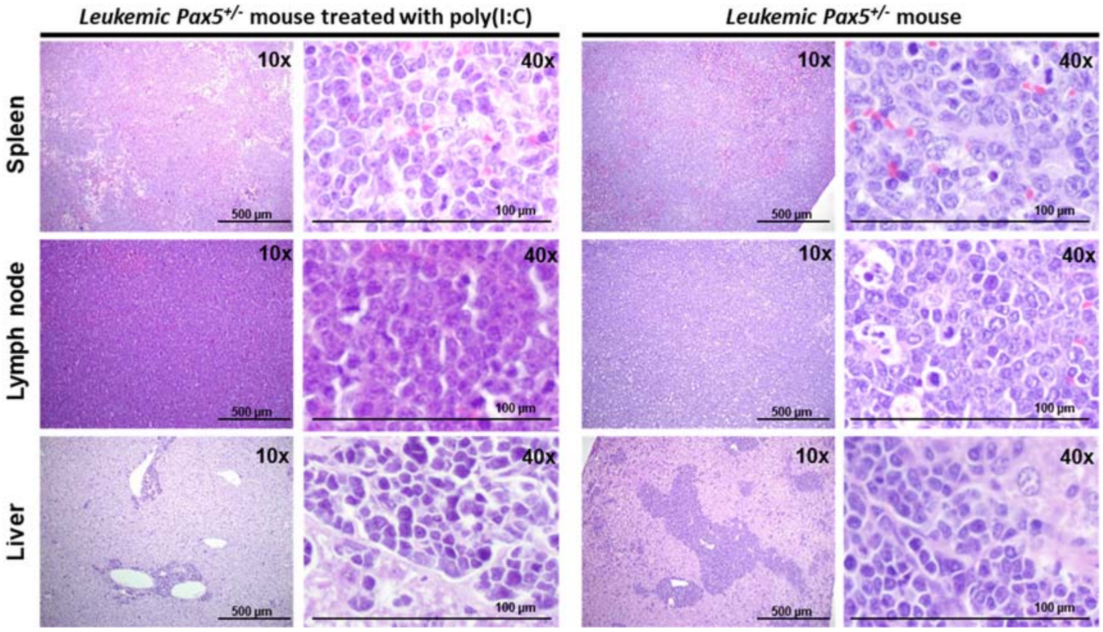

Supplementary Fig. S29: B-ALL in *Pax5*$^{+/-}$ mice treated or untreated with poly(I:C). Haematoxylin and eosin staining of tumour-bearing *Pax5*$^{+/-}$ mice treated or untreated with poly(I:C) showing infiltrating blast cells in the spleen, lymph nodes, and liver. Loss of normal architecture can be seen due to the infiltrating cells morphologically resembling lymphoblast. Magnification and the corresponding scale bar are indicated in each case.

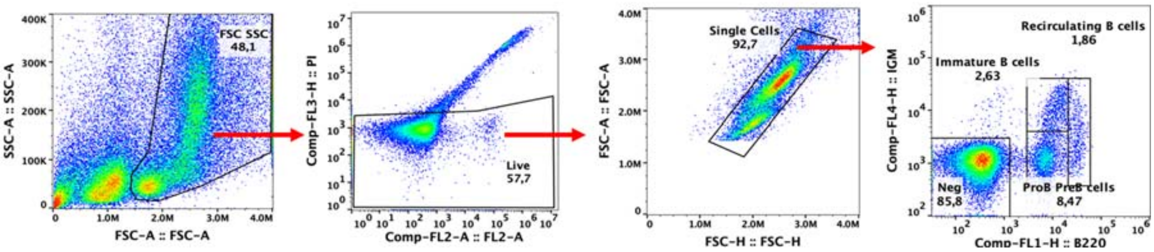

Supplementary Fig. S30: Gating strategy used in FACs analysis. Figure exemplifying the gating strategy used in all cytometric analysis. For each analysis, a total of at least 50,000 viable cells (PI-; Propidium iodide negative cells) were assessed. Singlets were selected prior gating strategy that is specific for each population. It is shown as an example, bone marrow cells stained with IgM-APC and B220-FITC. The same gating strategy has been used in all FACs analysis presented in Figure 2c, Figure 3c, Figure 4b, Figure 6b, Supplementary Fig. S3, Supplementary Fig. S6, Supplementary Fig. S9c, Supplementary Fig. S10, Supplementary Fig. S18, Supplementary Fig. S23, Supplementary Fig. S27, and Supplemental Figure 28.

REFERENCES

1 Juric, D. *et al.* Differential gene expression patterns and interaction networks in BCR-ABL-positive and -negative adult acute lymphoblastic leukemias *J Clin Oncol* **25**, 1341-1349, (2007).

2 Kohlmann, A. *et al.* Pediatric acute lymphoblastic leukemia (ALL) gene expression signatures classify an independent cohort of adult ALL patients *Leukemia* **18**, 63-71, (2004).

3 Linka, Y. *et al.* The impact of TEL-AML1 (ETV6-RUNX1) expression in precursor B cells and implications for leukaemia using three different genome-wide screening methods *Blood Cancer J* **3**, e151, (2013).

4 Liberzon, A. *et al.* The Molecular Signatures Database (MSigDB) hallmark gene set collection *Cell Syst* **1**, 417-425, (2015).

5 Mootha, V. K. *et al.* PGC-1alpha-responsive genes involved in oxidative phosphorylation are coordinately downregulated in human diabetes *Nat Genet* **34**, 267-273, (2003).

6 Subramanian, A. *et al.* Gene set enrichment analysis: a knowledge-based approach for interpreting genome-wide expression profiles *Proc Natl Acad Sci U S A* **102**, 15545-15550, (2005).
7 Green, M. R. *et al.* Signatures of murine B-cell development implicate Yy1 as a regulator of the germinal center-specific program *Proc Natl Acad Sci U S A* **108**, 2873-2878, (2011).
8 Revilla, I. D. R. *et al.* The B-cell identity factor Pax5 regulates distinct transcriptional programmes in early and late B lymphopoiesis *EMBO J* **31**, 3130-3146, (2012).
9 Schebesta, A. *et al.* Transcription factor Pax5 activates the chromatin of key genes involved in B cell signaling, adhesion, migration, and immune function *Immunity* **27**, 49-63, (2007).
