# Supplementary material for: Immune stress suppresses innate immune signaling in preleukemic precursor B-cells to provoke leukemia in predisposed mice
Source: Nat Commun. 2023 Aug 24;14:5159. doi: 10.1038/s41467-023-40961-z (PMC10449887; doi:10.1038/s41467-023-40961-z)
Supplement: Supplementary file 3 — Description of Additional Supplementary Files [file 41467_2023_40961_MOESM3_ESM.pdf]

DESCRIPTION OF ADDITIONAL SUPPLEMENTARY FILES for

Immune stress suppresses innate immune signaling in preleukemic precursor B cells to provoke leukemia in predisposed mice

Marta Isidro-Hernández M, et al.

SUPPLEMENTARY DATA 1: Differential abundance analysis of Taxa in *Sca1-BCR-ABLp190* mice compared with WT mice. All of them were housed in a pathogen-free facility. The differential LefSe analysis identified 74 taxonomic units with significant differences between stool DNA samples from Sca1-BCR-ABLp190 mice and WT mice. The taxonomic classification was done using the *SILVA* database to assign taxonomic labels to the V4-ASVs. Statistical analysis was done using LEfSe and conducted using α=1E-05 and minimum LDA score = 2. LDA, linear discriminant analysis. (*Table provided as an excel file*).

SUPPLEMENTARY DATA 2: Differential abundance analysis of Taxa in *Sca1-Lmo2* mice compared with WT mice. All of them were housed in a pathogen-free facility. The differential LefSe analysis identified 77 taxonomic units with significant differences between stool DNA samples from *Sca1-Lmo2* mice and WT mice. The taxonomic classification was done using the *SILVA* database to assign taxonomic labels to the V4-ASVs. Statistical analysis was done using LEfSe and conducted using α=1E-05 and minimum LDA score = 2. LDA, linear discriminant analysis. (*Table provided as an excel file*).

SUPPLEMENTARY DATA 3: Differential abundance analysis of Taxa in *Pax5*$^{+/-}$ mice compared with WT mice. All of them were housed in a pathogen-free facility. The differential LefSe analysis identified 127 taxonomic units with significant differences between stool DNA samples from *Pax5*$^{+/-}$ mice and WT mice. The taxonomic classification was done using the *SILVA* database to assign taxonomic labels to the V4-ASVs. Statistical analysis was done using LEfSe and conducted using α=1E-05 and minimum LDA score = 2. LDA, linear discriminant analysis. (*Table provided as an excel file*).

SUPPLEMENTARY DATA 4: Conventional Facility Health Monitoring Report. The table shows the pathogens tested to monitor the health status of the animals housed in the conventional facility during the time the animals have been studied.

SUPPLEMENTARY DATA 5: Gene list of differentially expressed genes in bone marrow B220^{+} cells from leukemic *Pax5*$^{+/-}$;*Myd88*$^{+/-}$ mice compared to control wild type proB and preB cells. (2463 genes-probesets with p-value<0.05 and fold change: FC<-2 or FC>2). Statistical analysis was done using ebayes limma method included in the TAC software. (*Table provided as an excel file*).

SUPPLEMENTARY DATA 6: Gene list of differentially expressed genes in bone marrow B220^{+} cells from leukemic *Pax5*$^{+/-}$;*Myd88*$^{+/-}$ mice compared to bone marrow B220^{+} cells from leukemic *Pax5*$^{+/-}$ mice. (1385 genes-probesets with p-value<0.05 and fold change: FC<-2 or FC>2). Statistical analysis was done using ebayes limma method included in the TAC software. (*Table provided as an excel file*).

SUPPLEMENTARY DATA 7: List of somatic mutations identified by Whole Genome Sequencing in 8 leukemic *Pax5*$^{+/-}$;*Myd88*$^{+/-}$ mice. Tumor DNA was derived from whole leukemic bone marrow or lymph nodes, while tail DNA of the respective mouse was used as reference germline material. (*Table provided as an excel file*).

SUPPLEMENTARY DATA 8: Gene list of differentially expressed genes in culture healthy *Pax5*$^{+/-}$ proB cells compared to healthy WT proB cells. (265 genes-probesets with p-value<0.05 and fold change: FC<-2 or FC>2). Statistical analysis was done using ebayes limma method included in the TAC software. (*Table provided as an excel file*).

SUPPLEMENTARY DATA 9: Gene list of differentially expressed genes in culture healthy *Pax5*$^{+/-}$;*Myd88*$^{+/-}$ proB cells compared to healthy *Pax5*$^{+/-}$ proB cells. (237 genes-probesets with p-value<0.05 and fold change: FC<-2 or FC>2). Statistical analysis was done using ebayes limma method included in the TAC software. (*Table provided as an excel file*).

SUPPLEMENTARY DATA 10: Gene list of differentially expressed genes in culture healthy *Pax5*$^{+/-}$;*Myd88*$^{+/-}$ proB cells compared to healthy WT proB cells. (327 genes-probesets with p-value<0.05 and fold change: FC<-2 or FC>2). Statistical analysis was done using ebayes limma method included in the TAC software. (*Table provided as an excel file*).

SUPPLEMENTARY DATA 11: Gene sets of human BCR-ABL+ and ETV6-RUNX1+ B-ALL samples[1-3] used for enrichment analysis. (*Table provided as an excel file*).
